# A Systematic Review of Foreign Language Listening Anxiety: Focus on the Theoretical Definitions and Measurements

**DOI:** 10.3389/fpsyg.2022.859021

**Published:** 2022-06-23

**Authors:** Suhe Ji, Xiaoqing Qin, Ke LI

**Affiliations:** ^1^School of Foreign Languages, Central China Normal University, Wuhan, China; ^2^School of Journalism and Communication, Wuhan University, Wuhan, China; ^3^Center for Studies of Media Development, Wuhan University, Wuhan, China

**Keywords:** foreign language listening anxiety, conceptual definition, measurement, instrument, systematic review, theoretical defining

## Abstract

A considerable amount of research on foreign language (FL) listening anxiety has emerged since 1986, yet a lack of sufficient attention on the conceptual definitions of FL listening anxiety and inappropriate employment of instruments to measure FL listening anxiety cause confusion in the research to a certain extent. This study presents a systematic review of 35 years of FL listening anxiety research. After initially searching 2,172 studies in 7 databases, 76 studies were identified for in-depth analysis. The results verified that the definitions of FL listening anxiety can be categorized into psychological, social, and situation-specific approaches, but the measure of FL listening anxiety was not only examined under these three approaches, but also additionally examined by sources of anxiety, learner characteristics, FL listening ability, and physiological factors. The results also showed that the definition of FL listening anxiety was not clear-cut nor that the measure was accurate, and to a great extent, the measure and the definition were inconsistent. This inconsistency can attribute to conceptual fuzziness in theoretical defining and casual utilization of scales without justification or explanation. We argue that future research needs to provide a tighter link between a more precise definition based on different situations and a valid measure of FL listening anxiety.

## Introduction

Listening comprehension has been considered an interactive process in which listeners positively construct meaning based on linguistic and non-linguistic information (e.g., Vandergrift and Goh, 2012). However, as one of the four language skills, listening has remained the least explored and was perceived as the most difficult language skill to learn (Vandergrift, 2007). This may be due to the complexity of listening. Different from written language, spoken language is transient, non-repetitive, and the meaning of certain words is incomprehensible until the whole sentence is presented. In this sense, listening can be highly anxiety-provoking. Despite its difficulties and anxiety eliciting, listening is the most frequently used language skill, and for most occasions, listening accompanies speaking in daily communication. Thus, listening is complex, dynamic, and hard work, which deserves more research and attention.

### The Theoretical Defining of Foreign Language Listening Anxiety

Before 2000, foreign language (FL) anxiety has established itself as one of the important variables responsible for the success or failure of FL learning, but the concept of FL listening anxiety was relatively new and conceptualized as a subtype of FL anxiety. A total of three approaches to defining FL anxiety were identified from extensive work before 2000 as the psychological, situation-specific, and social approaches (e.g., Horwitz et al., 1986; Young, 1990; MacIntyre and Gardner, 1994). First, the psychological approach conceptualized anxiety as feelings of tension and apprehension and heightened autonomic nervous system activity (Spielberger, 1971). Cognitive and affective components of anxiety were represented by worry and emotionality (see MacIntyre, 1995). Worry was assumed as a cognitive component of anxiety (Eysenck, 1979; Borkovec, 1985). Emotionality was largely associated with feelings of uneasiness, tension, and nervousness (Eysenck, 1979; Sarason, 1984). Different from the interference effects of worry, emotionality may not generate a negative effect, because when a task is simple, emotionality is facilitating anxiety; when a task is demanding and difficult, emotionality is debilitating anxiety. Second, the situation-specific approach claimed that foreign language anxiety was not a kind of subordination to other anxieties, but a distinct complex form of anxiety that some FL learners experienced in a classroom language learning setting (Horwitz et al., 1986), including speaking, learning, and listening (MacIntyre and Gardner, 1994). Third, the social approach to listening anxiety argued that when listeners held a negative belief about their listening ability and created a false impression that they must understand every single word they hear (Oxford, 1993; Vogely, 1998), they feel a sense of failure and frustration that may generate negative self-evaluation and affect listening comprehension. The social approach also believed that when decoding listening information, some listeners might fear “misinterpreting, inadequately processing and/or not being able to adjust psychologically to message sent by others” (Wheeless, 1975), and these fearful feelings that were regarded as the receiver apprehension can elicit listening anxiety (Ayres et al., 1995).

When the concept of FL listening anxiety was introduced in 2000 for the first time (Kim, 2005), it is recognized as a turning point in the study of FL listening anxiety. Extensive work on separating FL listening anxiety from general FL anxiety has been carried out at the phase between 2000 and 2014. The above three approaches examined the specific form of FL listening anxiety under a given perspective. For example, based on the results of factor analysis, the psychological approach considered FL listening anxiety as tension and worry over listening, the lack of confidence in listening (Kim, 2000), or as emotionality, worry, and anticipatory fear (Kimura, 2008). This psychological approach explores the common features of FL listening anxiety, which makes it more commonly taken; however, its inability to capture essence of FL listening anxiety in various situations seems to lead research toward the situation-specific approach. Chang (2008b) took the situation-specific perspective to identify FL listening anxiety in both general and test situations. Subsequently, a series of studies were conducted to testify listening anxiety in general and test situations (Chang, 2008a,^2010^; Chang and Read, 2008). But the general situation without a specified defining or explanation cannot demonstrate a specific role of FL listening anxiety in a given situation. Different from the psychological and situation-specific approaches to listening anxiety, the social approach claimed that FL listening anxiety was socially constructed because listening input was not limited to one-way listening, but was received from communicative and social events (Kimura, 2011).

Since 2014, the research on FL listening anxiety has recently flourished because researchers have begun to examine the relation between listening anxiety and other affective variables such as motivation (Bang and Hiver, 2016; Chow et al., 2018), self-efficacy (Fathi et al., 2020), meta-cognitive awareness (Xu and Huang, 2018), and listening achievement (Lee, 2016; Xu, 2017; Namaziandost et al., 2018). In terms of defining FL listening anxiety, the traditional three approaches are deeply embedded in the conceptualization of FL listening anxiety; however, the situation-specific approach has captured increasing attention in recent research (Lee, 2016; Jee, 2018; Wang and Cha, 2019). The apparent incongruence of the defining of the FL listening anxiety poses a challenge to the measure of FL listening anxiety. Mixed results are obtained because researchers adopt different approaches to define and measure FL listening anxiety with various instruments accordingly. For example, empirical research has yielded contradictory results of the relation between FL listening anxiety and listening achievements. Some studies showed that FL listening anxiety was a significant negative predictor of listening proficiency test (Bang and Hiver, 2016; Vafaee and Suzuki, 2019); however, some studies proved that FL listening anxiety was not a predictor for L2 listening test performance (Liu, 2016; Kim and Baek, 2017), and one study revealed that positive relation between listening anxiety and performance was found (*r* = 0.73, *p* < 0.001) (Naghadeh et al., 2014).

In sum, three approaches to FL listening anxiety form the theoretical frame of FL listening anxiety. Specifically, from a broader perspective, the psychological approach to FL listening anxiety suggests that listening anxiety manifests as worrisome and emotional feelings. The situation-specific approach considers *general* and *test* as two representative situations that can easily elicit listening anxiety. The social approach claims that *receiver apprehension* and *negative self-evaluation* are prone to elicit listening anxiety. This theoretical frame of FL listening anxiety is in accordance with what MacIntyre has defined general language anxiety: language anxiety was socially based, psychologically manifesting, and situation specifically constructed (MacIntyre, 1995). However, the multifaceted and complex, even contradictory theoretical defining of FL listening, makes the measure of FL listening vary to a great extent. Therefore, we put forward the first research question (RQ):

RQ1: How is FL listening anxiety defined in previous studies?

### The Measure of Foreign Language Listening Anxiety

There was no full scale to determine the existence or characteristics of FL listening anxiety before 2000; no doubt that research into FL listening anxiety was subordinate to general FL anxiety. Since Horwitz et al. (1986) developed the Foreign Language Classroom Anxiety Scale (FLCAS) to measure FL anxiety, the FLCAS has been widely used to explore the relationship between FL anxiety and skill-specific anxieties. Maybe it was the heavy weight on the importance of speaking and listening anxiety in FLCAS (Aida, 1994; Cheng et al., 1999) that leads to FLCAS being widely used to examine FL listening anxiety. However, it is not clear whether foreign language anxiety is a suite of anxieties; thereby, it might be inapplicable to apply a general scale to measure skill-specific anxiety.

The research on FL listening anxiety from 2000 to 2014 mainly focused on the original FL listening anxiety scale development, followed by research on validation and revision of these scales. The first domain-specific full-scale targeting FL listening anxiety, Foreign Language Listening Anxiety Scale (FLLAS), was developed by Kim (2000). Until 2014, several original scales aimed at specifically identifying FL listening anxiety have been developed and re-analyzed by subsequent studies. These originally developed scales include the psychological-based FLLAS (Kim, 2000), the situation-specific-based FLLAS (Chang, 2008b), and a scale without detailed dimensionality information (Elkhafaifi, 2005). Kim’s FLLAS (Kim, 2000) characterized as psychometric properties was revised by Kimura (2008) and Yamauchi (2014b), and the results of factor analyses yielded different component structures of the original FLLAS, which indicates the factors extracted from the original scale, and subsequent duplication studies are neither valid nor stable. The questionable validity of these original FLLAS may partially attribute to the inconsistency between the conceptual defining and the measure of FL listening anxiety. For example, Kim (2000) took the social approach to define FL listening anxiety as receiver apprehension; however, factor analysis of FLLAS revealed that FL listening anxiety was measured from the psychological perspective as tension, worry, and lack of confidence. In addition, the revised version of Kim’s (2000) FLLAS showed that FL listening anxiety was measured by sources of listening anxiety, such as factors related to listening material, listeners’ cognitive process, and factors other than the material (Yamauchi, 2014b). Elkhafaifi (2005) adopted the situation-specific approach to develop another commonly used listening anxiety scale, but the subsequent factor analysis of the scale indicates that the measure is tapping into the source of listening anxiety, because FL listening anxiety was measured mainly by state anxiety, self-belief, and listening decoding skills (Zhang, 2013). This inconsistency between the theoretical defining and measure of FL listening anxiety widens the gap between what researchers intend to measure and what they actually measure.

In the recent years, research on FL listening anxiety has shifted attention from scale development, validation, and revision to other domains. These recently focused research domains involved the complex relation between listening anxiety and other individual difference variables such as motivation, strategy, and working memory (Chow et al., 2018; Namaziandost et al., 2018), the causal relationship between listening anxiety and achievement (Zhang, 2013; Vafaee and Suzuki, 2019), and instructional applications to reduce listening anxiety (Fathi et al., 2020). However, the lack of discussion regarding the inconsistency between the theoretical defining and measure of FL listening anxiety makes the selection of a variety of scales uncontroversial. When scales are chosen without justification, it may lead to the measure invalid to a great extent. In fact, the mismatch between the conceptual defining and measure of FL listening anxiety prevails since the initial research on FL listening to scale development and revision research on listening anxiety; this mismatch and lack of justification and explanation of scale selection make it difficult to choose a proper instrument to assess FL listening anxiety validly. For example, some researchers took the psychological approach (Rezaabadi, 2016), or the situation-specific approach (Xu and Huang, 2018; Liu and Xu, 2021) to define FL listening anxiety; however, they did not employ a situation-specific-based scale to measure FL listening anxiety in a test situation; rather, they chose a scale without detailed dimensionality information to measure listening anxiety in a high-stake test situation. Thus, researchers differ widely on the measurements of FL listening anxiety, which yields mixed outcomes in previous research. It is questionable about the extent to which these measurements probe into the exact FL listening anxiety that researchers mean to examine. Therefore, we put forward the following research questions:

RQ2: How is FL listening anxiety measured in previous studies?

RQ3: Are the measurements consistent with the theoretical defining of FL listening anxiety in these studies?

Ideally, the theoretical defining of FL listening anxiety is consistent with its measurement, which is in accord with corresponding research objectives. When different measurements cannot examine what researchers intend to measure, several factors might be under question. First, there seems a great possibility that studies on the same or similar themes tend to adopt the same scale to examine FL listening anxiety, even though they differ on the defining of FL listening anxiety. Second, there may be a geographical preference in adopting a certain scale to tap into FL listening anxiety. For example, Kim (2000) developed FLLAS based on the investigation of Korean university students’ FL listening anxiety; it seems likely that Korean researchers might adopt Kim’s scale to examine Korean FL learners’ listening anxiety. Thus, the same first language (L1) of participants and/or researchers probably influences the choice of certain FL listening anxiety scales. In addition, participants’ major, target language, or age might affect the different adoptions of FL listening anxiety instruments. In addition, due to the complex and intertwining relation between theoretical approaches to FL listening anxiety, some researchers may blur the distinction between these approaches, which may contribute to the mismatch between the theoretical defining and measurement of FL listening anxiety. However, the defining of FL listening anxiety from different approaches is one of the most important factors that may directly influence the selection of certain instruments to measure FL listening anxiety. According to the above possibilities that might contribute to the ineffectiveness of the measurement on FL listening anxiety, we put forward the following research questions:

RQ4: What are the methodological characteristics of FL listening anxiety in previous research?

RQ5: What factors influence the selection of different FL listening anxiety instruments?

### Study Approach

The FL research has witnessed a growth in interest in the construct of FL listening anxiety over the last three decades. The rich literature reveals a clear trend to probe the nature of FL listening anxiety and explore the development of FL listening anxiety over time. However, several unsettled issues hindered the progress of research. Among these issues, the variety and fuzzy defining of FL listening anxiety and ways to measure the multidimensional construct are the main two challenges. Previous research has proved that it was important to carefully define anxiety and choose an appropriate measure in the study of anxiety (Scovel, 1978). This finding was considered as a turning point in the study of anxiety and language learning by Horwitz (2010), because it pointed out that imprecision in the theoretical defining and measure of anxiety produced inconsistent results. Therefore, it is necessary to explicitly investigate the existing conceptual defining and measures of FL listening anxiety as decades have passed since the first study of FL listening anxiety. Taking into account these previous considerations, this systematic review aimed to synthesize the research on the theoretical defining and measures of FL listening anxiety and probe into the relationship between the definitions and measures of FL listening anxiety.

Based on the aims of this systematic review, the study approach adopted for data analysis was a narrative content analysis. A systematic review is a particular kind of review that uses explicit and systematic methods to identify studies that meet pre-specified eligibility criteria, with the aim of answering specific research questions (Moher et al., 2015). Different from a traditional narrative review, a systematic review requires a thorough and objective search of all the potentially relevant studies within resource limits (Higgins and Green, 2011). After the comprehensive collection of data, the following data analysis of a systematic review may be a narrative content analysis, or a meta-analysis. The former analysis involves subjective analysis with focuses on critical assessments of included studies and discussion of characteristics and findings; the latter analysis is the statistical combination of results (Higgins and Green, 2011). However, it is inappropriate to use meta-analysis when the outcomes of included studies are diverse (Higgins and Green, 2011). Thus, due to the diversity of defining and measures of FL listening anxiety, this systematic review adopted a narrative content analysis approach for data analysis, which was a commonly used method in the FL research (e.g., Macaro et al., 2018; Zhang, 2019; Hiver et al., 2021).

## Methodology

### Search Strategy

In this review, we used some of the search strategies recommended by Cooper et al. (2019). First, we conducted a scoping search to have a rough understanding of the scale and scope of the literature (Siddaway et al., 2018; Cooper et al., 2019).

Second, based on the research questions, we identified the concept of FL listening anxiety. In this review, we defined FL listening anxiety as tension and worry of miscomprehension of spoken language in FL learning situations that is operationalized and measured through psychological, social, or situation-specific approaches. This FL listening anxiety may be generated as the consequence of listening performance, or as a cause of listening performance. Although the original intention of identifying the concept of the research topic was to develop the search strategy, the outcomes concepts were not included in the search strategy because it was difficult to capture the various outcomes (Cooper et al., 2019). In this review, we aimed to investigate how FL listening anxiety was defined and measured, but in the vast abundant of literature, how FL listening anxiety was defined and measured can be described in many ways and may not be addressed or listed in an abstract; thus, the conceptual defining of FL listening anxiety was not included in the search strategy.

Third, we identified search terms. According to Cochrane handbook for systematic reviews, when searching for potential studies for a systematic review, search terms should be viewed with special caution, because some available terms might not correspond to the terms that the searchers wished to use (Higgins and Green, 2011). In this review, we found that some search terms (e.g., *listening stress*) were not appropriate to identify studies related to the subject of this review, because they identified studies that were irrelevant to this review. For example, the term *listening stress* mostly identified studies on listening to music to reduce psychobiological or physiological stress. Furthermore, the search terms related to methodology should be excluded to ensure sensitivity (comprehensive search) and specificity (maintaining relevance) (Fernández-Martín et al., 2021); thus, search terms such as *define* and *measure* should be avoided in this review. However, a search term that was found in the search strategies in published research synthesis can be identified for the present research synthesis (Cooper et al., 2019). Therefore, the search term *listening anxiety* that was used in the meta-analysis study of Zhang (2019) was identified as the search term for this review. To balance striving for sensitivity and specificity, search terms constructed by Boolean operators were used in this review. The Boolean operator OR enables to expand the search results; the AND operator narrows down the search scope, and NOT operator will exclude some search results (Cooper et al., 2019). Search terms were adjusted to accommodate databases due to different search functions of these databases. Therefore, literature searches were conducted by topic and/or abstract. For example, the query used in the search of Education Resources of Information Center (ERIC) was *listening* AND *anxiety*.

Fourth, we conducted the main searches. Because it is common to follow literature search guidelines suggested by Plonsky (2015) when conducting a systematic review in applied linguistics research (e.g., Brown, 2016; Uchihara et al., 2019; Yanagisawa and Webb, 2021), we conducted the main searches by following these guidelines and examined the following the most common electronic databases, internet, and citation indexes: Education Resources of Information Center (ERIC), Linguistics and Language Behavior Abstracts (LLBA), PsycINFO, Academic Search Premier, ProQuest Dissertations, Google Scholar, and Web of Science (Plonsky, 2015). Based on the guidelines by Plonsky (2015), we included unpublished doctoral dissertations and journal articles of FL listening anxiety. The time span was set from 1986 to 2021 (Zhang, 2019).

In the last phase, we also performed a manual search of highly relevant journals for potential studies to identify any records that were not captured by the search strategies. The whole literature search began in 2021 and ended in 8 May 2021.

After searching and identifying potential studies, we screened all relevant studies according to the eligibility criteria. To ensure consistency and rigor, we followed The PRISMA Statement (Page et al., 2021), one of the main guidelines and checklists for reporting systematic reviews and meta-analyses (Siddaway et al., 2018).

### Eligibility Criteria

After locating the primary studies, a list of inclusion and exclusion criteria was applied to define the boundaries of the review (Siddaway et al., 2018; Cooper et al., 2019). Studies that met the inclusion criteria were to be included in the final analysis, and studies that met the exclusion criteria were to be excluded.

The inclusion criteria were the following:

• Academic publication must range from 1986 to 2021. The reason why 1986 was determined as the inception was that it was the year when Horwitz et al. (1986) developed the FLCAS, and specified FL anxiety as a unique learning process anxiety not merely a composite of other anxieties (Zhang, 2019). Based on the theory and measurement, the four skill-specific anxieties were identified and reported as distinct language skill anxieties.
• Only research articles that include the definitions or precise measurements of FL listening anxiety were included in the final sample pool.
• The research subjects had to be FL learners.
• The study had to be written in English.

Exclusion criteria were applied during the selection process:

• Studies were conducted before 1986.
• Studies recruited teachers as research subjects.
• Studies without definitions and measurements of FL listening anxiety were excluded.
• Studies that focused on investigating listening anxiety in the mother tongue were excluded.
• Systematic reviews and meta-analysis studies were excluded.

### Selection and Data Collection Process

**FIGURE 1:**
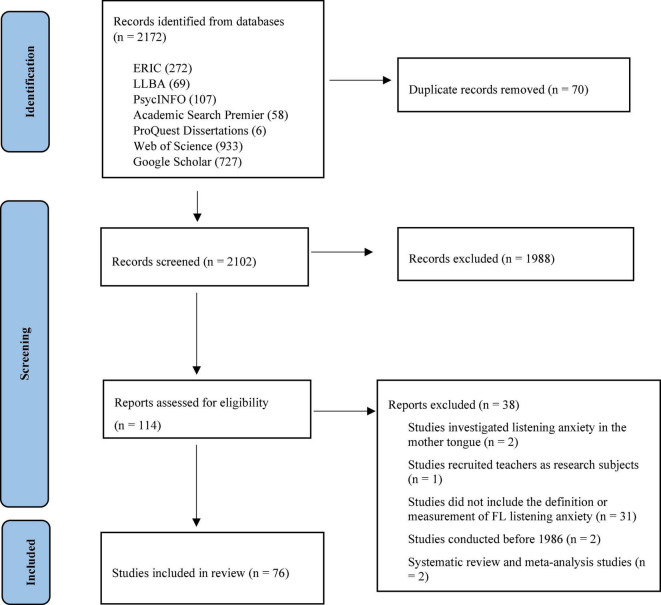
PRISMA flowchart of study selection.

The first database search identified 2,172 potentially eligible studies: 272 from ERIC, 69 from LLBA, 107 from PsycINFO, 58 from Academic Search Premier, 6 from ProQuest Dissertations, 727 from Google Scholar, and 933 from Web of Science. After the removal of 70 duplicates, 2,102 studies were screened by titles and abstracts. A total of two authors assessed the titles and abstracts independently and they discussed disagreements until differences were resolved. After the titles and abstracts screening, 1,988 studies were excluded. These two authors examined the remaining 114 studies for full-text against the above eligibility criteria. A total of 38 studies were eliminated: two studies investigating listening anxiety in the mother tongue, one study targeting teachers’ listening anxiety, 31 studies without definition nor measurements of FL listening anxiety, two studies before 1986, and two systematic review and meta-analysis studies. Accordingly, 76 studies were included in the qualitative synthesis, including 73 journal articles, and 3 dissertations. Figure 1 shows the flow diagram of study selection.

### Data Extraction

A 13-item pre-piloted data extraction form was used to extract data. This data extraction form was based on Plonsky’s suggested categories for coding in L2 research (Plonsky, 2015) and previous instruments for assessing methodological quality (Plonsky and Gass, 2011; Plonsky, 2013; Hiver et al., 2021). The data extraction included the following categories: (a) study identification (e.g., author, year of publication, country, publication type), (b) study context (e.g., sample size, major, age, participants’ first language, participants’ target language), and (c) study characteristics (e.g., method, analysis technique). In addition, some categories in the coding scheme were based on qualitative analysis (Mackey and Gass, 2012), such as *definition* and *factor analysis*, and this part of the qualitative analysis would be presented later. A pilot data extraction was conducted first between two raters by coding 5 randomly chosen studies, so that the coding scheme could be revised. A total of two authors extracted all the data of the total sampled 76 studies. Interrater reliability scores were calculated using Cohen’s Kappa. The following Table 1 shows that agreement achieved above strong agreement among different coding categories (McHugh, 2012). A total of two raters negotiated any discrepancies by re-examining the studies and discussing together until consensus was reached.

**TABLE 1:** Reliability of coding.

Category	Kappa
Sample	0.95
Major	0.98
Age	0.97
First language	0.98
Second language	1.00
Definition	0.89
Method	0.94
Theme	0.92
Scale	0.96
Analysis technique	0.82

### Data Analysis

In line with the research questions of this review, several analytical techniques were applied. To answer the first research question, which was concerning the theoretical defining of FL listening anxiety in previous research, a template analysis was conducted. According to Hesse-Biber (2018), a template analysis was a top-down approach to summarize major themes. It began with a set of *a priori* themes that came out of previous research; if there were no suitable prior themes, a new theme could be created. In this study, the *priori* themes were identified from the research literature. In the literature, FL listening anxiety language anxiety was defined under the psychological, social, and situation-specific approaches. In addition, this theoretical frame was in accord with MacIntyre’s assumption that language anxiety was psychologically, socially, and situation-specifically constructed (MacIntyre, 1995). Having excluded 3 studies that did not specify precise definitions in the entire pool of 76 studies, we extracted the definitions of FL listening anxiety in the remaining 73 studies. Then, we coded these original definitions based on the theoretical frame of FL listening anxiety. Based on the coding of definitions, we calculated the frequencies and percentage of different approaches to define FL listening anxiety.

To answer the second research question that addressed the measure of FL listening anxiety, we first analyzed FL listening anxiety scales development, validation, adoption, and revision; then, we employed a template analysis to analyze the dimensionality of FL listening anxiety scales. Specifically, having excluded qualitative studies (*n* = 9) and a study targeting measuring general language anxiety (*n* = 1) in the initial pool of 76 studies, we analyzed the remaining 66 quantitative studies for the descriptive analysis of the measurements of FL listening anxiety. Following the descriptive analysis, a top-down template analysis was employed to examine the construct of FL listening anxiety scales based on 15 studies involving factor analysis of scales. According to the literature review of previous research, the theoretical frame that FL listening anxiety was psychologically, socially, and situation specifically constructed was introduced as a set of *priori* themes. If there were no suitable prior themes, the template analysis allowed to introduce a new theme by a bottom-up analysis, which involved a coding procedure with three levels (Yan and Horwitz, 2008). In the Level 1 coding, authors conducted grounded reading of the original quotations of the raw material (Hesse-Biber, 2018). In the Level 2 coding, patterns were identified based on the grounded reading. In the Level 3 coding, new themes were specified from Level 2. Based on the coding of dimensionality of various scales, we calculated the frequencies and percentage of different approaches to measure FL listening anxiety.

To answer the third research question, whether the measures were consistent with the theoretical conceptions of FL listening anxiety, we calculated frequencies and percentages with which different measures were associated with the definitions. Specifically, having excluded three studies without precise definitions in the pool of 66 quantitative studies, we collected 63 studies with both definitions and measurements of FL listening anxiety to analyze the extent to which various measurements examined the theoretical definitions of FL listening anxiety. Based on the first two research questions, different types of theoretical defining of FL listening anxiety and the constructs of FL listening anxiety scales were obtained. In this phase of analysis, descriptive analysis was used to present the frequencies and percentages of various instruments related to different types of definitions of FL listening anxiety.

With regard to the main focus of the fourth research question, 66 quantitative studies created a study pool for the descriptive analysis of the methodological characteristics of these studies. This phase of analysis involved calculating frequencies and percentages of different sample size, major, age, participants’ first languages (L1), participants’ target languages, methods, and analysis techniques.

Turning to the last research question, which addressed factors influencing the selection of different types of FL listening anxiety instruments, a categorical regression analysis was employed to examine the influence of different types of variables in relation to the selection of instruments. A total of 63 studies involving both the definitions and measurements of FL listening anxiety were retained for the categorical regression analysis. Because major, L1, definition, and theme are categorical variables, categorical regression with optional scaling (CATREG) was performed. In this phase of categorical regression analysis, the dependent variable was the selection of various scales, and independent variables were (a) major of participants, (b) participants’ first language, (c) the theoretical definitions of FL listening anxiety, and (d) main research themes that reflect the primary focus of FL listening anxiety research.

## Results

### Defining Foreign Language Listening Anxiety

The qualitative portion of this systematic review investigated how FL listening was defined in previous research. The extracted original definitions are shown in Supplementary Material 1, and the results of the template analysis of definitions are detailed in Supplementary Material 2. The results showed that 28, 21, and 16 studies adopted the psychological, social, and situation-specific approach to define FL listening anxiety, respectively, and eight studies adopted more than one approach as the construction of FL listening anxiety (refer to Supplementary Material 2). Table 2 shows that 10 studies (13.7%) adopted a cognitive approach to defining FL listening anxiety as a kind of worry, which was considered as a psychological barrier affecting listening comprehension tasks, and 27 studies (37.0%) defined FL listening anxiety as affective emotionality, which characterized FL listening anxiety as tenseness, irritation, frustration, apprehension, nervousness, and uneasiness. The social approach featured FL listening anxiety either as receiver apprehension (*k* = 20, 27.4%) or negative self-evaluation (*k* = 5, 6.8%). The situation-specific approach to FL listening anxiety took learning settings into account when defining FL listening anxiety and specified two major situations where FL learners experienced FL listening anxiety: general situations (*k* = 19, 26.0%) and the test situation (*k* = 6, 8.2%). The former situations were general language learning situations, ranging from classroom language context to communication situations. The latter situation was associated with the high-stakes test situation that had great potential of anxiety-provoking.

**TABLE 2:** Domains of FL listening anxiety.

Themes	Sub-themes	K	%	Studies
Psychological	Worry	10	13.7	MacIntyre and Gardner, 1994; MacIntyre, 1995; Cebreros, 2003; Kimura, 2008; Mohammadi Golchi, 2012; Afshar and Hamzavi, 2014; Yamauchi, 2014a; Li, 2015; Namaziandost et al., 2018; Kaivanpanah et al., 2020
	Emotionality	27	37.0	MacIntyre, 1995; Mills et al., 2006, 2007; Noro, 2006, 2010; Bekleyen, 2009; Wang, 2010; Yang, 2010; Kimura, 2011; Atasheneh and Izadi, 2012; Pae, 2013; Valizadeh and Alavinia, 2013; Chen and Lin, 2014; Choi and Chon, 2014; Yamauchi, 2014b; Li, 2015; Bang and Hiver, 2016; Liu, 2016; Pan, 2016; Rezaabadi, 2016; Otair and Abd Aziz, 2017; Xu, 2017; Halat and Özbay, 2018; Polat and Eristi, 2019; Vafaee and Suzuki, 2019; Fathi et al., 2020; Niimoto, 2021
Social	Receiver apprehension	20	27.4	Kim, 2000, 2002; Chang, 2010; Wang, 2010, 2016; Kimura, 2011, 2017; Kiliç and Uçkun, 2012; Mohammadi Golchi, 2012; Capan and Karaca, 2013; Serraj and Noordin, 2013; Zhai, 2015; Ali, 2017; Berber and Gönen, 2017; Yassin and Razak, 2017; Namaziandost et al., 2018; Angellia and Listyani, 2019; Babakhouya and Elkhadiri, 2019; Ranto Rozak et al., 2019; Nurkhamidah, 2020
	Negative self-evaluation	5	6.8	MacIntyre, 1995; Vogely, 1998; Xu, 2011; Tsai, 2013; Chow et al., 2018
Situation-specific	General	19	26.0	Horwitz et al., 1986; MacIntyre, 1995; Chang, 2008b; Bekleyen, 2009; Zhang, 2013; Brunfaut and Révész, 2014; Movahed, 2014; Moghadam et al., 2015; Rahimi and Soleymani, 2015; Lee, 2016; Liu, 2016; Jee, 2018; Namaziandost et al., 2018; Kutuk et al., 2019; Vafaee and Suzuki, 2019; Wang and Cha, 2019; Hutapea et al., 2020; Kaivanpanah et al., 2020; Hamid and Idrus, 2021
	Test	6	8.2	Chang, 2008a,b; Chang and Read, 2008; Xu and Huang, 2018; Kutuk et al., 2019; Vafaee and Suzuki, 2019

*K, number of studies.*

The results above revealed the most frequently adopted approach to define FL listening anxiety was the psychological approach, followed by the social approach and the situation-specific approach. Moreover, eight studies adopted more than one approach, which made the theoretical defining more confusing. These results suggest that there is no clear-cut boundary among the three dimensions of FL listening anxiety. In terms of the first research question, the results showed that FL listening anxiety was defined under the psychological, social, and situation-specific approaches.

### Measuring Foreign Language Listening Anxiety

The measure of FL listening anxiety involved a variety of scales development and adoption. First, six studies originally developed scales to measure FL listening anxiety. Among them, five studies targeted at identifying FL listening anxiety from different perspectives (Kim, 2000; Elkhafaifi, 2005; Mills et al., 2006; Chang, 2008b; Kutuk et al., 2019); however, one study aimed at examining general foreign language anxiety (Horwitz et al., 1986). But this general foreign language anxiety scale was utilized to measure FL listening anxiety directly due to its heavy weight on the importance of speaking and listening anxiety (Aida, 1994; Cheng et al., 1999; Pae, 2013). Second, with regard to the adoption of various instruments, we found that 30 studies adopted or modified Kim’s (2000) FLLAS; 15 studies employed Elkhafaifi’s (2005) FLLAS to examine FL listening anxiety; eight studies adopted or modified Horwitz et al.’s (1986) FLCAS as the instrument to measure FL listening anxiety (refer to Supplementary Material 3). Therefore, in terms of citation of original FL listening anxiety scales, the most cited top three scales were Kim’s (2000) FLLAS, Elkhafaifi’s (2005) FLLAS, and Horwitz et al.’s (1986) FLCAS.

To answer how FL listening anxiety was measured, we analyzed the dimensionalities of the 15 scales involved factor analysis. First, we extracted factors from these studies as the raw materials (refer to Supplementary Material 2). Then, a top-down template analysis was employed to examine the dimensionality of these scales. Table 3 shows that the dimensions of various FL listening anxiety scales shared three sub-components: the *psychological*, *social*, and *situation-specific* construct, which were consistent with theoretical frame of FL listening anxiety. However, results also showed that four new themes were created by the bottom-up coding procedure. These four new themes included *sources of anxiety*, *learner characteristics*, *FL listening ability*, and *physiological* approach. *Sources of FL listening anxiety* referred to the external arousal factors that can elicit FL listening anxiety. *Learner characteristics* referred to individual differences associated with dimensions of enduring personal characteristics when learning a second/foreign language. *FL listening ability* referred to FL learners’ skills or language competence to perform various FL listening tasks. The *physiological* approach referred to explicit physiological symptoms of the anxiety experience that may result in certain avoidance behaviors. These newly generated themes were more abstract theoretical constructs of FL listening anxiety, which indicates that the measure of FL listening anxiety diverges from the theoretical defining of FL listening anxiety.

**TABLE 3:** The dimensionality of FL listening anxiety scales and the measurements of FL listening anxiety under various approaches.

Themes	Sub-themes	*K*	%	Coding	Studies
Psychological	Worry	32	48.5	Worry over English, anticipatory fear, cognitive, factors related to listeners’ cognitive processes; listening anxiety; lack of confidence in listening	Kim, 2000, 2011; Kimura, 2008; Bekleyen, 2009; Wang, 2010, 2016; Kiliç and Uçkun, 2012; Mohammadi Golchi, 2012; Capan and Karaca, 2013; Pae, 2013; Serraj and Noordin, 2013; Tsai, 2013; Afshar and Hamzavi, 2014; Choi and Chon, 2014; Movahed, 2014; Yamauchi, 2014b; Li, 2015; Rahimi and Soleymani, 2015; Zhai, 2015; Bang and Hiver, 2016; Berber and Gönen, 2017; Cheng, 2017; Halat and Özbay, 2018; Jee, 2018; Namaziandost et al., 2018; Babakhouya and Elkhadiri, 2019; Kutuk et al., 2019; Ranto Rozak et al., 2019; Fathi et al., 2020; Hutapea et al., 2020; Hamid and Idrus, 2021; Niimoto, 2021
	Emotionality	33	50.0	Tension over English, emotionality, affective, listening anxiety	Kim, 2000, 2011; Kimura, 2008; Bekleyen, 2009; Wang, 2010, 2016; Kiliç and Uçkun, 2012; Mohammadi Golchi, 2012; Capan and Karaca, 2013; Pae, 2013; Serraj and Noordin, 2013; Tsai, 2013; Zhang, 2013; Afshar and Hamzavi, 2014; Movahed, 2014; Li, 2015; Rahimi and Soleymani, 2015; Zhai, 2015; Bang and Hiver, 2016; Liu, 2016; Berber and Gönen, 2017; Xu, 2017; Halat and Özbay, 2018; Jee, 2018; Namaziandost et al., 2018; Xu and Huang, 2018; Babakhouya and Elkhadiri, 2019; Kutuk et al., 2019; Ranto Rozak et al., 2019; Wang and Cha, 2019; Fathi et al., 2020; Hutapea et al., 2020; Hamid and Idrus, 2021
Social	Receiver apprehension	4	6.1	Low confidence in comprehending spoken English	Chang, 2008a,b, 2010; Chang and Read, 2008
	Negative self-evaluation	0	0	/	/
Situation-specific	General	10	15.2	Taking English listening courses as a requirement	Horwitz et al., 1986; MacIntyre and Gardner, 1994; Cebreros, 2003; Chang, 2008a,b, 2010; Chang and Read, 2008; Atasheneh and Izadi, 2012; de Dios Martínez Agudo, 2013; Yassin and Razak, 2017
	Test	5	7.6	Testing anxiety, worrying about test difficulty	Chang, 2008a,b, 2010; Chang and Read, 2008; Choi and Chon, 2014
Sources of anxiety	Material	4	6.1	Factors related to the material itself, the control of listening sources, task-focused apprehension	Kimura, 2011, 2017; Yamauchi, 2014b; Niimoto, 2021
	Environment	3	4.5	Environmental elements, factors other than the material, the control of listening sources, ascribed meaning to listening, individual and environmental elements	Yamauchi, 2014b; Polat and Eristi, 2019; Niimoto, 2021
Learner characteristics	Self-belief	5	7.6	Self-belief	Zhang, 2013; Liu, 2016; Xu, 2017; Xu and Huang, 2018; Wang and Cha, 2019
	Self-efficacy	3	4.5	Self-efficacy; self-focused apprehension	Yang, 2010; Kimura, 2011, 2017
FL listening ability	Language skills	5	7.6	FL listening decoding skills, decoding-skills	Zhang, 2013; Liu, 2016; Xu, 2017; Xu and Huang, 2018; Wang and Cha, 2019
	Prior knowledge	3	4.5	Concern about insufficient prior knowledge	Wang, 2010, 2016; Choi and Chon, 2014
Physiological	Somatic	3	4.5	Physiological, somatic, somatic anxiety	Yang, 2010; Cheng, 2017; Kutuk et al., 2019
	Behavioral	2	3.0	Behavioral	Li, 2015; Cheng, 2017

The wide divergence between the measure and theoretical defining of FL listening anxiety may be associated with the replication of FL listening anxiety scales or re-analysis of the dimensionality of FL listening anxiety scales. For example, it was found that Kim’s (2000) FLLAS was re-analyzed the dimensionality of FL listening anxiety by Kimura (2008, 2011) and revised by Wang (2010) and Yamauchi (2014b). A total of two new themes were generated from the re-analysis of dimensionality: *sources of anxiety* and *FL listening ability*. In addition, Kim’s (2000) original FLLAS was adapted by Wang (2010), and a new construct of FL listening anxiety scale was created, i.e., *FL listening ability*. In addition, in Yamauchi’s (2014b) verification study of Kim (2000), a new theme *sources of anxiety* was formed.

Table 3 also presents an overview of how the studies we reviewed measured FL listening anxiety. Studies that employed psychologically based scales to measure FL listening anxiety were clearly the majority, with 32 studies (48.5%) measuring worry of FL listening anxiety and 33 studies (50.0%) measuring emotionality of FL listening anxiety. The rarest were studies that employed socially focused scales to measure FL listening anxiety, because only four studies (6.1%) measured FL listening anxiety as receiver apprehension but none of study measured FL listening anxiety as negative self-evaluation. A total of 15 studies adopted situation-specific-based scales to measure FL listening anxiety, with 10 studies (15.2%) measuring general listening anxiety and five studies (7.6%) measuring listening test anxiety. Other studies that employed various approaches to measure FL listening anxiety were only minimally present; for example, seven studies measured FL listening anxiety under the approach of the sources of anxiety; eight studies measured FL listening under the approaches of learner characteristics and FL listening ability, respectively, and five studies measured FL listening anxiety under the physiological approach.

Thus, we concluded that FL listening anxiety was measured in various ways in previous quantitative research. Specifically, six original scales were developed to examine FL listening anxiety, which were adopted or adapted by subsequent studies later. The analysis of the dimensionality of FL listening anxiety measurements revealed that FL listening anxiety was measured from psychological, social, and situation-specific approaches; additionally, FL listening anxiety was also examined by the sources of anxiety, learner characteristics, FL listening ability, and physiological approach.

### The Relation Between the Theoretical Defining and Measurements of Foreign Language Listening Anxiety

Based on the results of dimension analysis of FL listening anxiety scales (RQ2), we coded the measure of FL listening anxiety, which is depicted in Supplementary Material 3. Then, frequency percentages were calculated to show the extent to which the measure is consistent with the defining of FL listening anxiety (refer to Table 4). Table 4 shows that 11 studies (17.5%) used psychologically focused scales to measure FL listening anxiety that was defined under the psychological approach. However, the remaining studies utilized other scales unrelated to the psychological construction of FL listening anxiety. These scales involved situation-specific-based scales (9.5%), scales aimed at exploring sources of anxiety (4.8%), scales focused on learner characteristics (3.2%), FL listening ability focused scales (1.6%), physiologically based scales (3.2%), and scales with unknown dimensionality (11.1%).

**TABLE 4:** The relation between the theoretical conceptions and measurements of FL listening anxiety.

Theoretical conceptions	Measurements	*K*	%
Psychological	Psychological	11	17.5
	Situation-specific	6	9.5
	Sources of anxiety	3	4.8
	Learner characteristics	2	3.2
	FL listening ability	1	1.6
	Physiological	2	3.2
	Unknown	7	11.1
Social	Psychological	10	15.9
	Social	1	1.6
	Situation-specific	2	3.2
	Learner characteristics	3	4.8
	Sources of anxiety	2	3.2
	FL listening ability	2	3.2
	Unknown	2	3.2
Situation-specific	Psychological	9	14.3
	Social	3	4.8
	Situation-specific	4	6.3
	Learner characteristics	2	3.2
	FL listening ability	3	4.8
	Physiological	1	1.6
	Unknown	3	4.8
Mixed	Psychological	5	7.9
	FL listening ability	2	3.2
	Unknown	2	3.2

Concerning defining and measuring FL listening anxiety from the social approach, only one study (1.6%) examined FL listening anxiety under the social approach utilizing the corresponding scale. Other studies defined FL listening anxiety from the social approach but measured it by the psychologically based scales (15.9%), situation-specific-based scales (3.2%), learner characteristics focused scales (4.8%), scales targeting sources of anxiety (3.2%), FL listening ability focused scales (3.2%), and scales with unknown dimensionality (3.2%).

Turning to the studies of both defining and measuring FL listening anxiety under the situation-specific approach, the results found that only four studies (6.3%) used a proper scale to examine FL listening anxiety that was defined under the situation-specific approach. The improper scales included psychologically based scales (14.3%), socially focused scales (4.8%), the learner characteristics focused scales (3.2%), the FL listening ability based scales (4.8%), physiologically based scales (1.6%), and scales with unknown dimensionality (4.8%).

Studies with mixed approaches to the definition measured FL listening anxiety most frequently by psychologically based scales (7.9%), followed by the FL listening ability based scales (3.2%), and scales with unknown dimensionality (3.2%).

To sum up, there were only 16 studies (25.4%) that employed proper measurements to examine FL listening anxiety based on the theoretical conceptions. This result indicates that the extent of the operational measurements has not achieved the ideal expectation. In other words, to a great extent, the measurements have not examined what researchers intend to. The majority of measurements neglect the theoretical analysis of the scales and roughly adopt a scale to measure FL listening anxiety.

### The Methodological Characteristics of Foreign Language Listening Anxiety Studies

Research question 4 concerned the methodological characteristics of the quantitative studies. Detailed information of sample sizes, major, age, L1, L2, themes, method, and analysis technique is provided in Supplementary Material 3. As shown in Table 5, the descriptive results of sample size showed that quite the same number of studies sampled between 50 and 100 (28.8%), 100 and 200 participants (27.3%), and 200 and 500 participants (25.8%), but a few studies selected more than 500 participants (9.1%). It suggests that studies tend to rely on large samples to obtain the data on FL listening anxiety. This finding was in line with the claim that larger samples and probing into individual differences were closely linked (Brown et al., 2018). With regard to the academic major of participants, the largest proportion of the participants in the sampled studies were non-English major students (33.3%), followed by English major participants (27.3%). However, previous studies showed that academic major did make a difference in the FL listening anxiety scores (Kim, 2000; Kimura, 2008). The ages of participants were featured a majority of university students (75.8%), followed by secondary school (12.1%) participants. But younger learners’ FL listening anxiety as well as the relation between anxiety and achievement remained relatively unexplored (Horwitz, 2001). As noted, the majority of participants’ L1 was Chinese, followed by Japanese and Turkish. In terms of L2, it was found that English was the dominant target language being learned, remarkably accounting for 89.4%, whereas other languages learned as L2 (e.g., Spanish, Arabic, Korean, and Turkish) were found in one single study. These results suggest that the current outstanding status of the L2 learning situation is featured the Asian and Middle East learners learning English as the target language. However, with so complicate L1 background, the selection of scales to measure FL listening anxiety may relate to L1 background information. Therefore, it seems possible that participants’ academic major and L1 may influence the selection of different instruments to measure FL listening anxiety.

**TABLE 5:** Participant characteristics and study context.

	*K*	%
***Sample size***		
1 < N ≤ 50	4	6.1
50 < N ≤ 100	19	28.8
100 < N ≤ 200	18	27.3
200 < N ≤ 500	17	25.8
N > 500	6	9.1
Multiple sample	2	3.0
***Major***		
Non-English major	22	33.3
English major	18	27.3
Mixed	3	4.5
Unclear	18	27.3
No major	5	7.6
***Age***		
Primary school	1	1.5
Secondary school	8	12.1
University	50	75.8
Mixed age groups	4	6.1
Unclear	3	4.5
***First language (L1)***		
Arabic	3	4.5
Chinese	19	28.8
Egyptian	1	1.5
English	4	6.1
Indonesian	2	3.0
Iranian	5	7.6
Japanese	7	10.6
Korean	6	9.1
Malay	1	1.5
Persian	4	6.1
Spanish	3	4.5
Turkish	7	10.6
Mixed	4	6.1
***Second language (L2)***		
English	59	89.4
Spanish	1	1.5
Arabic	1	1.5
French	3	4.5
Turkish	1	1.5
Korean	1	1.5

*K, number of studies.*

The in-depth review was concerned with methods, different themes associated with FL listening anxiety, and the analysis technique employed to analyze different forms of data. Table 6 shows detailed information on the above concerns. Regarding themes of research on FL listening anxiety, the topics that had been explored varied. Of the 66 studies included in the sample, the most frequently conducted research themes were the relation between FL listening anxiety and listening achievement (*k* = 18), which was parity with the number of studies concerning the relation between FL listening anxiety and affective variables (*k* = 18). As noted, 16 studies focused on the measurements of FL listening anxiety, which mainly explored the constructs of FL listening anxiety, followed by the development and/or validation of FL listening anxiety scales. In addition, many studies (*k* = 14) tapped into sources and/or effects of FL listening anxiety. Other frequently examined themes were the measure of different levels of FL listening anxiety among L2 learners with various L1 backgrounds (*k* = 10), the relation between FL listening anxiety and psychological variables, such as intelligence, working memory (*k* = 8), and the relation between FL listening anxiety and instruction applications (*k* = 6). A relatively small proportion of studies explored the relation between FL listening anxiety and other anxieties, i.e., four skill-based anxieties and general classroom FL learning anxiety (*k* = 5). The above results showed vast broad research themes concerning the primary focus of FL listening anxiety. These research themes might influence the choice of scales, because the same category of research may employ the same or similar scale to investigate FL listening anxiety.

**TABLE 6:** In-depth review of studies by theme, method, and analysis technique.

	*K*	%
***Themes***		
Relation between FLLA and listening achievement	18	27.3
Different levels of FLLA	10	15.2
Relation between FLLA and other anxieties	5	7.6
Relation between FLLA and psychological variables	8	12.1
Sources and/or effects of FLLA	14	22.7
Measurements of FLLA	16	24.2
Relation between FLLA and instructional applications	6	10.6
Relation between FLLA and affective variables	18	27.3
***Methods***		
Quantitative	61	92.4
Mixed	5	7.6
***Analysis techniques***		
Descriptive analysis	5	7.6
Conventional inferential statistics	38	57.6
Advanced multivariate statistics	23	34.8

*K, number of studies; FLLA, foreign language listening anxiety.*

As noted, the methods employed by the included studies were dominated by the feature of quantitative studies (92.4%). The methods adopted by the quantitative studies covered from questionnaire survey (*k* = 54), experimental design (*k* = 11), to longitudinal design (*k* = 1). Because the majority of studies were quantitative research, it was found that conventional inferential statistical analyses (57.6%) and advanced multivariate statistical analyses were the top two frequently adopted methods (34.8%). The conventional inferential statistical analyses included *t*-tests (*k* = 11), analyses of variance (ANOVA) (*k* = 18), correlations (*k* = 25), chi-square tests (*k* = 2), and linear regression analysis (*k* = 2). A total of 23 studies employed advanced multivariate statistical analyses, which covered multivariate analysis of variance (MANOVA) (*k* = 2), analysis of covariance (ANCOVA) (*k* = 6), factor analysis (*k* = 15), multiple regression (*k* = 11), structural equation model SEM analysis (*k* = 6), and cluster analysis (*k* = 1). The minority of qualitative studies used coding and analysis methods (*k* = 1); quite many studies (*k* = 5) adopted descriptive analysis.

### Factors Influencing Different Selections of Foreign Language Listening Anxiety Instruments

Turning to the influence of methodological characteristics on the employment of various scales, the results of categorical regression analysis revealed that four factors can explain almost 63% (*R*^2^ = 0.63) of the variance in the employment of various scales to measure FL listening anxiety. The analysis of variance reported in Table 7 illustrated that an F statistic of 2.07 with *p* < 0.05, together with an R square value of 0.63, which suggested that the model performed well. From the standardized regression coefficients (refer to Table 8), it can be concluded that L1 was the only variable in the model that could predict the employment of different scales to measure FL listening anxiety (*p* < 0.001). This result suggested that the selection of scales to measure FL listening anxiety is not based on the theoretical definition of FL listening anxiety. This robust result also echoed the qualitative analysis of research question 3 that the measurements of FL listening anxiety were not consistent with the conceptual definitions.

**TABLE 7:** ANOVA table.

Model	Sum of squares	Df.	Mean square	*F*	*p*
Regression	39.72	28	1.42	2.07	0.02
Residual	23.28	34	0.69		
Total	63.00	62			

**TABLE 8:** Categorical regression coefficients.

	Standardized coefficients				Correlations		
Variables	Beta	Standard error	*F*	*p*	Zero-order	Partial	Part
Major	0.36	0.24	2.30	0.08	0.12	0.49	0.34
L1	0.78	0.25	9.46	0.00	0.63	0.77	0.73
Definition	0.26	0.27	0.95	0.42	0.25	0.39	0.26
Theme	0.36	0.29	1.53	0.18	0.11	0.48	0.33

## Discussion

### The Theoretical Defining of Foreign Language Listening Anxiety

**FIGURE 2:**
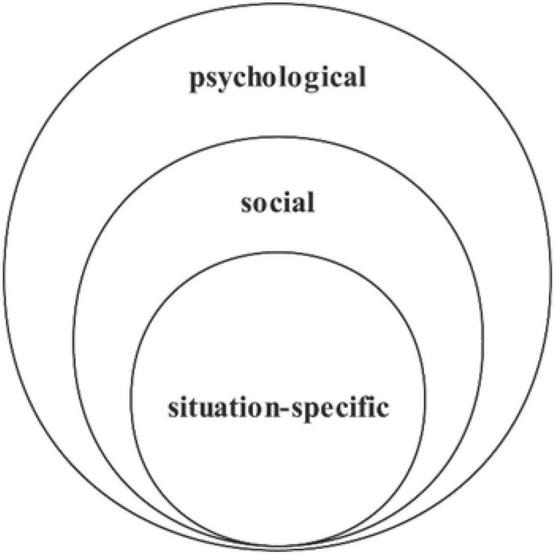
The macro-, meso-, and microsystem of FL listening anxiety.

First, both previous research and the results of our review have shown that FL listening anxiety was defined under three approaches: the psychological, social, and situation-specific approaches (Wheeless, 1975; Eysenck, 1979; Horwitz et al., 1986; MacIntyre, 1995), and this review further showed that the psychological approach to FL listening anxiety was found to be the most frequently adopted, followed by the social approach and the situation-specific approach. These three approaches serve as macro-, meso-, and microsystem levels to examine FL listening anxiety (refer to Figure 2). The tripartite notion can be regarded as the theoretical model of FL listening anxiety. The macrosystem level is the psychological approach, which is responsible for psychological mechanisms of all kinds of anxiety including FL listening anxiety. The mesosystem level is the social approach, which manifests the effects of the receiver and evaluative anxiety on listeners in social communication contexts. The microsystem level is the situation-specific approach that defines listening anxiety in distinct FL learning settings. This tripartite notion explains the phenomenon that there is no clear-cut boundary in terms of theoretical defining of FL listening anxiety. In addition, the result that the most frequently adopted approach was the macro-approach suggests that FL listening anxiety is not specifically well defined. In other words, so many studies seemed to be clouded by the fuzzy boundary of the theoretical defining of FL listening anxiety, leading them to take the broadest way to define it. Moreover, as this systematic review shows, some studies took more than one approach to define FL listening anxiety, and only 19 (26.0%) studies provided a clear definition or an operational definition instead of mentioning a definition in the introduction or background information. These findings indicate that a large number of studies we reviewed adopt unclear-cut, unfocused, and non-transparent definitions of FL listening anxiety in the applied linguistic contexts.

Second, although the agreement on the situation specificity of foreign language anxiety was supported by many researchers (Horwitz et al., 1986; MacIntyre and Gardner, 1989; MacIntyre, 1992), situations were not clearly defined. Some terms used to specify FL listening situations were generalized (e.g., *situation-specific*), imprecise (e.g., *listening-related tasks*), and vague (e.g., *situations which need listening, FL listening situations*, and *when engaging in L2 listening*). Some notions of situation specificity did not distinguish FL listening situations from *FL classroom*, *general situations*, and *test situations*, because FL classroom does not equate to general situations, and test situations are not common general situations. Such conceptual fuzziness and unclear-cut boundary in defining situations no doubt affect the measure of FL listening anxiety.

### The Measure of Foreign Language Listening Anxiety

One positive trend in studies we reviewed was the inclusion of various self-report measurements to examine FL listening anxiety. It was suggested that the way of self-report of internal feelings did have an advantage in precision than the physiological way of testing physiological reactions when tapping into different measurements of anxiety (Scovel, 1978). However, one negative trend in these studies was distantly related scales being adapted to develop a new FL listening anxiety scale. For example, Foreign Language Reading Anxiety Scale, Achievement Emotions Questionnaire, Speaking Anxiety Questionnaire, and Mathematics Anxiety Scale were adapted when developing a new FL listening anxiety scale (Elkhafaifi, 2005; Mills et al., 2006; Chang, 2008b; Kutuk et al., 2019). It indicates that these original scale development studies are neither theoretically well-constructed, nor based on the theoretical defining of FL listening anxiety. The original intention of the research design is inclined to measure the common and stable psychological anxiety, with which FL listening anxiety can be measured in a listening situation. Such intention inevitably leads to a more extensive measurement of FL listening anxiety and an undesired outcome. Another negative trend in studies we reviewed was newly emerged dimensions of FL listening anxiety scales widened the gap of valid measurements of FL listening between the original scale development studies and the subsequent studies. The results of dimensionality analysis showed that four new themes were generated by the bottom-up process of the template analysis. To a great extent, the former three new themes (i.e., *sources of anxiety*, *learner characteristics*, and *FL listening ability*) can be considered as factors related to sources of FL listening anxiety. However, sources of anxiety only account for the cause of FL listening anxiety, rather than the components of listening anxiety. It is imprecise and inappropriate to measure an intrinsic variable (i.e., FL listening anxiety) with an extrinsic indicator (i.e., sources of FL listening anxiety) (Scovel, 1978). The fourth theme (i.e., *physiological* approach) was a physiological indicator; however, the physiological indicator is more accurate to measure physical activities than to measure anxiety, because anxiety is a psychological construct in nature. Therefore, these new themes of the subsequent studies make the gap between the theoretical defining and measure of FL listening anxiety wider.

No doubt that one of the primary functions of a systematic review is to describe and evaluate research methodology, and to provide empirically based suggestions, thus to inform future research in a given domain. With respect to the measurement of FL listening anxiety, we found less work on developing new scales under the situation-specific approach. The development of technology and media has expanded FL learning from traditional academic settings to a variety of informal and incidental learning situations; thus, new situations in which FL listening anxiety is easily elicited should be taken into consideration when developing a new scale (Pekrun et al., 2011). Because the interplay between FL listening anxiety in academic settings and FL listening anxiety in informal learning situations is a new direction toward which FL listening anxiety research should move.

### The Inconsistency Between Theoretical Defining and Measurements of Foreign Language Listening Anxiety

This review found that only a small proportion of studies (25.4%) measured FL listening anxiety using appropriate scales. The mismatch between the measurement and theoretical defining of FL listening anxiety can largely attribute to conceptual fuzziness in theoretical defining and casual utilization of scales without justification or explanation. Such inconsistency between the defining and measure of FL listening anxiety no doubt introduces a major threat to the validity of measures and the findings they produce. For example, a negative relationship between FL listening anxiety and listening test was found in some studies (Bang and Hiver, 2016; Vafaee and Suzuki, 2019), and no relationship and positive relationship were found in other studies (Naghadeh et al., 2014; Liu, 2016; Kim and Baek, 2017). These findings indicate that the inconsistent results found in FL listening anxiety and listening performance may attribute to imprecision in the theoretical defining and measurements of FL listening anxiety. This result was in line with the study of Scovel (1978) who concluded that incomplete correlations between anxiety and measures of language proficiency stem from inaccurate defining and measure of anxiety. He found that inconsistent results of relationship between anxiety and language achievement were observed because various studies defined different types of anxiety (e.g., facilitating-debilitating anxiety, state-trait anxiety) and measured anxiety with different ways (e.g., behavioral tests, self-report of internal feelings, and physiological tests). In addition, Young (1991) argued that whether the defining and measure of anxiety were consistent was often overlooked. Our review also found that a large proportion of studies we reviewed adopted the most frequently used scale (i.e., Kim’s (2000) FLLAS), but these studies defined FL listening anxiety from various perspectives although they employed the same scale to measure FL listening. Accordingly, the inconsistency between the conceptual defining and measurement of FL listening anxiety confuses the study on FL listening anxiety further and sheds light on the questionable validity of some research.

Looking more closely at the inconsistency issue, the results of categorical regression analysis showed that variables, such as participants’ major, definition, and research themes, cannot predict different selections of scales; however, participants’ L1 background can influence the selection of scales. On the one hand, the statistical findings echoed the quantitative analysis of the inconsistency between the defining and measure of FL listening anxiety. On the other hand, the influence of participants’ L1 on the selection of scales reveals that cultural distance and cognate linguistic distance may influence the selection of a scale to measure FL listening anxiety. For example, Japanese researchers would most likely use Kim’s (2000) FLLAS to examine Japanese EFL learners’ FL listening anxiety (Kimura, 2008; Yamauchi, 2014b); meanwhile, Spanish researchers tended to employ Horwitz et al.’s (1986) FLCAS to measure Spanish EFL Learners’ listening anxiety (Cebreros, 2003; de Dios Martínez Agudo, 2013); in other words, the different selection of instruments might be attributed to a narrower cultural distance between Japan and Korea, and between Spain and America. These findings suggest that cognate linguistic distance and cultural distance between the source language and the target language should be taken into great consideration when measuring FL listening anxiety with different instruments. The findings also reveal that previous research on FL listening anxiety is the lack of adequate theoretical basis, and the selection of instruments is too broad and imprecise. Therefore, it is important to identify FL listening anxiety with a precise and clear-cut boundary of defining, and extreme caution should be used when measuring FL listening anxiety based on corresponding theoretical defining. Sorting out the precise nature and measurement can inform pedagogy and help FL learners learn better in a non-threatening environment.

## Conclusion and Implications

The systematic analysis approach provided a rather robust method for investigating how FL listening anxiety has been defined and measured since 1986. The purpose of this review was to take stoke of work in this field and examine whether the conceptual definitions of FL listening were consistent with measurements, and further probe into reasons for the mismatch between the theoretical defining and measurements. We found that FL listening anxiety was defined and measured under three approaches: the psychological, social, and situation-specific approaches; we also found that FL listening anxiety was additionally measured by the sources of anxiety, learner characteristics, learners’ FL listening ability, and the physiological approach. Further thematic analysis and categorical regression analysis showed that the theoretical defining of FL listening anxiety was inconsistent with measurements, and definitions cannot influence the selection of instruments to measure FL listening anxiety, but participants’ L1 can affect the selection of instruments. This systematic review highlights the need of using precise defining with consistent measurement of FL listening anxiety in future research and the importance of understanding and clarifying the abstract theoretical constructs of FL listening anxiety on the deepening the insights of empirical studies, on directions for the advancing measurements, and on the educational practices.

Further pedagogical implications of the findings of this review are of great importance. First, it is necessary to clarify the conceptual definition and assess the prevailing measurements of FL listening anxiety, so as to help instructors and researchers better understand how different types of FL listening anxiety affect listening achievement and other learning variables. Second, the findings of this review suggest that a new scale based on various situations which are prone to elicit FL listening anxiety should be developed. With such a new scale, instructors may find it useful to distinguish different types of FL listening anxiety, so that instructors can develop specific instructional strategies to reduce FL listening anxiety under the situation-specific approach. For example, in the FL classroom situation, instructors can provide various, comprehensible, and authentic input to increase FL listening practice (Young, 1991). Instructors should encourage FL learners to have growth mindsets that language competence can be cultivated (Lou and Noels, 2016). Such belief can motivate learners to persist and feel less anxious in challenging situations (Lou and Noels, 2020). In the listening test situations, instructors should assist learners to take effective strategies such as progressive relaxation, deep breathing, or meditation to overcome the tense in the listening tests (Oxford, 1990). In outside the classroom situations, teachers may guide learners to conduct more extensive listening, because it can help students process spoken language with ease and less worry (Liu, 2006; Renandya and Farrell, 2011). Third, instructors should pay special attention to high-anxiety students who need more emotional support and trust from teachers. It is crucial for instructors to endeavor to build a secure environment and establish a trust-worthy relationship between students and teachers. Only in such environment can seeking help and collaborative learning take place.

There are still areas to be pressed ahead in future research. First, more attention should be paid to the consistency of the definition and measurement in the follow-up study to reduce the bias of research results as much as possible. Second, considering the diversity of situations where FL listening anxiety may be elicited, FL listening anxiety should be defined based on the variety of situations. Additionally, a new situation-based instrument that is consistent with the theoretical defining of FL listening anxiety should be developed, so as to make the corresponding research more precise and accurate, and help learners more accurately adjust their learning plans in anxious learning situations. We do hope that future work will lead to a richer range of research on FL listening anxiety.

## Data Availability Statement

The raw data supporting the conclusions of this article will be made available by the authors, without undue reservation.

## Author Contributions

SJ performed identification and screening studies, coded all studies, and drafted the entire manuscript. XQ resolved conflict, revised the subsequent draft, and proofread the entire manuscript. KL designed the systematic review study, screened and coded all studies, and conducted the statistical analysis of this study. All authors contributed to this study, read and approved the submitted version.

## Conflict of Interest

The authors declare that the research was conducted in the absence of any commercial or financial relationships that could be construed as a potential conflict of interest.

## Publisher’s Note

All claims expressed in this article are solely those of the authors and do not necessarily represent those of their affiliated organizations, or those of the publisher, the editors and the reviewers. Any product that may be evaluated in this article, or claim that may be made by its manufacturer, is not guaranteed or endorsed by the publisher.

## References

[B1] Afshar H. S. Hamzavi R. (2014). The relationship among reflective thinking, listening anxiety and listening comprehension of Iranian EFL learners: does proficiency make a difference? *Issues Lang. Teach.* 3 237–261.

[B2] de Dios Martínez Agudo J. (2013). An investigation into Spanish EFL learners’. *Anxiety. Rev. Bras. Linguística Apl.* 13 829–851.

[B3] Aida Y. (1994). Examination of Horwitz, Horwitz, and cope’s construct of foreign language anxiety: the case of students of Japanese. *Mod. Lang. J.* 78 155–168. 10.1111/j.1540-4781.1994.tb02026.x

[B4] Ali M. A. K. (2017). English language anxiety: development and validation of a brief measure. *Int. J. Psychol. Educ. Stud.* 4 42–53.

[B5] Angellia, and Listyani. (2019). Freshmens anxiety in an intensive listening class: a qualitative study. *Educ. Res. Rev.* 14 443–457. 10.5897/err2018.3624

[B6] Atasheneh N. Izadi A. (2012). The role of teachers in reducing/increasing listening comprehension test anxiety: a case of Iranian EFL learners. *Engl. Lang. Teach.* 5 178–187.

[B7] Ayres J. Wilcox A. K. Ayres D. M. (1995). Receiver apprehension: an explanatory model and accompanying research. *Commun. Educ.* 44 223–235. 10.1080/03634529509379013

[B8] Babakhouya Y. Elkhadiri Y. (2019). An investigation of the relation between neuroticism and english language listening anxiety. *Adv. Lang. Lit. Stud.* 10:112. 10.7575/aiac.alls.v.10n.4p.112

[B9] Bang S. Hiver P. (2016). Investigating the structural relationships of cognitive and affective domains for L2 listening. *Asian-Pacific J. Second Foreign Lang. Educ.* 1 1–19. 10.1186/s40862-016-0013-8

[B10] Bekleyen N. (2009). Helping teachers become better english students: causes, effects, and coping strategies for foreign language listening anxiety. *System* 37 664–675.

[B11] Berber G. Gönen S. ÝK. (2017). How do high and low anxious FL listeners employ FL listening comprehension strategies? exploring student perspectives. *J. Qual. Res. Educ* 5 90–108. 10.14689/issn.2148-2624.1.5c3s4m

[B12] Borkovec T. D. (1985). Worry: a potentially valuable concept. *Behav. Res. Ther.* 23 481–482. 10.1016/0005-7967(85)90178-0 4026778

[B13] Brown D. (2016). The type and linguistic foci of oral corrective feedback in the L2 classroom: a meta-analysis. *Lang. Teach. Res.* 20 436–458. 10.1177/1362168814563200

[B14] Brown A. V. Plonsky L. Teimouri Y. (2018). The use of course grades as metrics in L2 research: a systematic review. *Foreign Lang. Ann.* 51 763–778. 10.1111/flan.12370

[B15] Brunfaut T. Révész A. (2014). The role of task and listener characteristics in second language listening. *TESOL Q.* 49 141–168.

[B16] Capan S. A. Karaca M. (2013). A comparative study of listening anxiety and reading anxiety. *Procedia Soc. Behav. Sci.* 70 1360–1373. 10.1016/j.sbspro.2013.01.198

[B17] Cebreros A. M. O. (2003). Measuring language anxiety perceived by Spanish university students of English. *Bells Barc. Engl. Lang. Lit*. 12. Available online at: https://raco.cat/index.php/Bells/issue/view/6981

[B18] Chang A. C. S. (2008a). Listening strategies of L2 learners with varied test tasks. *TESL Can. J.* 25 1–26.

[B19] Chang A. C. S. (2008b). Sources of listening anxiety in learning English as a foreign language. *Percept. Mot. Skills* 106 21–34. 10.1108/eb003506 18459352

[B20] Chang A. C. S. (2010). Second-language listening anxiety before and after a 1-yr. intervention in extensive listening compared with standard foreign language instruction. *Percept. Mot. Skills* 110 355–365. 10.2466/PMS.110.2.355-365 20499548

[B21] Chang A. C.-S. Read J. (2008). Reducing listening test anxiety through various forms of listening support. *TESL-EJ* 12 1–25.

[B22] Chen W. Lin W.-Y. (2014). A study on the relationship of english listening comprehension to linguistic, cognitive and affective variables among taiwanese elementary school students. *Accent. Asia* 7 1–27.

[B23] Cheng Y. S. (2017). Development and preliminary validation of four brief measures of L2 language-skill-specific anxiety. *System* 68 15–25. 10.1016/j.system.2017.06.009

[B24] Cheng Y. S. Horwitz E. K. Schallert D. L. (1999). Language anxiety: differentiating writing and speaking components. *Lang. Learn.* 49 417–446. 10.1111/0023-8333.00095

[B25] Choi J. Y. Chon Y. V. (2014). Listener anxiety and listening strategies on multiple-choice items of EFL learners. *J. Korea English Educ. Soc.* 13 21–53. 10.18649/jkees.2014.13.4.21

[B26] Chow B. W. Y. Chiu H. T. Wong S. W. L. (2018). Anxiety in reading and listening English as a foreign language in Chinese undergraduate students. *Lang. Teach. Res.* 22 719–738. 10.1177/1362168817702159

[B27] Cooper H. Hedges L. V. Valentine J. C. (2019). *The Handbook of Research Synthesis and Meta-Analysis.* New York, NY: Russell Sage Foundation.

[B28] Elkhafaifi H. (2005). Listening comprehension and anxiety in the Arabic language classroom. *Mod. Lang. J.* 89 206–220. 10.1111/j.1540-4781.2005.00275.x

[B29] Eysenck M. W. (1979). Anxiety, learning, and memory: a reconceptualization. *J. Res. Pers.* 13 363–385. 10.1016/0092-6566(79)90001-1

[B30] Fathi J. Derakhshan A. Torabi S. (2020). The effect of listening strategy instruction on second language listening anxiety and self-efficacy of Iranian EFL learners. *SAGE Open* 10 1–13. 10.1177/2158244020933878

[B31] Fernández-Martín F. D. Romero-Rodríguez J. M. Marín-Marín J. A. Gómez-García G. (2021). Social and emotional learning in the Ibero-American context: a systematic review. *Front. Psychol.* 12:738501. 10.3389/fpsyg.2021.738501 34659053 PMC8516388

[B32] Halat S. Özbay M. (2018). the examination of listening anxiety level of the students who learn Turkish as a foreign language. *Univers. J. Educ. Res.* 6 1–10.

[B33] Hamid T. M. H. T. A. Idrus F. (2021). A correlational study of the english listening and speaking anxiety in rural areas. *Engl. Lang. Teach.* 14 9–19.

[B34] Hesse-Biber S. (2018). Gender differences in psychosocial and medical outcomes stemming from testing positive for the BRCA1/2 genetic mutation for breast cancer: an explanatory sequential mixed methods study. *J. Mix. Methods Res.* 12 280–304. 10.1177/1558689816655257

[B35] Higgins J. P. Green S. (2011). *Cochrane Handbook for Systematic Reviews of Interventions.* London: The Cochrane Collaboration.

[B36] Hiver P. Al-Hoorie A. H. Vitta J. P. Wu J. (2021). Engagement in language learning: a systematic review of 20 years of research methods and definitions. *Lang. Teach. Res.* 10.1177/13621688211001289

[B37] Horwitz E. K. (2001). Language anxiety and achievement. *Annu. Rev. Appl. Linguist.* 21 112–126.

[B38] Horwitz E. K. (2010). Foreign and second language anxiety. *Lang. Teach.* 43 154–167. 10.1017/S026144480999036X

[B39] Horwitz E. K. Horwitz M. B. Cope J. (1986). Foreign language classroom anxiety. *Mod. Lang. J.* 70 125–132. 10.5840/ajs2012281-29

[B40] Hutapea S. C. Adnan A. Marlina L. (2020). The correlation between EFL’s students listening motivation with listening anxiety in intermediate listening classes. *J. English Lang. Teach.* 9 520–530. 10.24036/jelt.v9i3.44054

[B41] Jee M. J. (2018). Four skill-based foreign language anxieties: learners of Korean in Australia. *Linguist. Res.* 35 23–45. 10.17250/khisli.35.201809.002

[B42] Kaivanpanah S. Mohammad Alavi S. Al-Shammari H. (2020). Examining the effect of listening strategy instruction on EFL Iraqi learners’ listening anxiety. *Arab World English J.* 62–75. 10.24093/awej/elt2.4

[B43] Kiliç M. Uçkun B. (2012). Listening text type as a variable affecting listening comprehension anxiety. *English Lang. Teach.* 6 55–62. 10.5539/elt.v6n2p55

[B44] Kim J. (2000). *Foreign Language Listening Anxiety: A Study of Korean Students Learning English.* Unpublished Doctoral dissertation. Austin, TX: The University of Texas.

[B45] Kim J. (2002). Affective reactions to foreign language listening retrospective interviews with Korean EFL students. *Lang. Res.* 38 117–151.

[B46] Kim J. (2005). The reliability and validity of a foreign language listening anxiety scale. *Korean J. English Lang. Linguist.* 5 231–235.

[B47] Kim J.-S. (2011). Korean EFL learners’ listening anxiety, listening strategy use, and listening proficiency. *English Lang. Lit. Teach.* 17 101–124.

[B48] Kim M. Baek S. (2017). The structural relationship of factors in Korean fifth graders’ L2 listening proficiency. *English* 30 337–364.

[B49] Kimura H. (2008). Foreign language listening anxiety: its dimensionality and group differences. *JALT J.* 30:173. 10.37546/jaltjj30.2-2

[B50] Kimura H. (2011). *A Self-Presentational Perspective On Foreign Language Listening Anxiety* Ph. D Thesis. Philadelphia, PA: Temple University Graduate Board.

[B51] Kimura H. (2017). Foreign language listening anxiety: a self-presentational view. *Int. J. List.* 31 142–162. 10.1080/10904018.2016.1222909

[B52] Kutuk G. Putwain D. W. Kaye L. Garrett B. (2019). Development and validation of a new multidimensional language class anxiety scale. *J. Psychoeduc. Assess.* 38 649–658. 10.1177/0734282919875881

[B53] Lee S. P. (2016). Computer-detected attention affects foreign language listening but not reading performance. *Percept. Mot. Skills* 123 33–45. 10.1177/0031512516657337 27371638

[B54] Li H. (2015). A study of EFL listening anxiety in a test setting. *Int. J. English Linguist.* 5 106–114. 10.5539/ijel.v5n2p106

[B55] Liu M. (2006). Anxiety in Chinese EFL students at different proficiency levels. *System* 34 301–316. 10.1016/j.system.2006.04.004

[B56] Liu M. (2016). Interrelations between foreign language listening anxiety and strategy use and their predicting effects on test performance of high- and low-proficient Chinese University EFL learners. *Asia Pac. Educ. Res.* 25 647–655.

[B57] Liu M. Xu H. (2021). Testing effects of foreign language listening anxiety on Chinese University students’. English Listening Test Performance. *Front. Psychol.* 12:701926. 10.3389/fpsyg.2021.701926 34322069 PMC8310933

[B58] Lou N. M. Noels K. A. (2016). Changing language mindsets: implications for goal orientations and responses to failure in and outside the second language classroom. *Contemp. Educ. Psychol.* 46 22–33. 10.1016/j.cedpsych.2016.03.004

[B59] Lou N. M. Noels K. A. (2020). Breaking the vicious cycle of language anxiety: growth language mindsets improve lower-competence ESL students’ intercultural interactions. *Contemp. Educ. Psychol.* 61:101847. 10.1016/j.cedpsych.2020.101847

[B60] Macaro E. Curle S. Pun J. An J. Dearden J. (2018). A systematic review of English medium instruction in higher education. *Lang. Teach.* 51 36–76. 10.1017/S0261444817000350

[B61] MacIntyre P. D. (1992). *Anxiety And Language Learning From A Stages Of Processing Perspective. Unpublished doctoral dissertation.* Ontario, TO: University of Western Ontario.

[B62] MacIntyre P. D. (1995). How does anxiety affect second language learning? A reply to sparks and Ganschow. *Mod. Lang. J.* 79 90–99. 10.1111/j.1540-4781.1995.tb05418.x

[B63] MacIntyre P. D. Gardner R. C. (1989). Anxiety and second-language learning: toward a theoretical clarification. *Lang. Learn.* 39 251–275. 10.1111/j.1467-1770.1989.tb00423.x

[B64] MacIntyre P. D. Gardner R. C. (1994). The subtle effects of language anxiety on cognitive processing in the second language. *Lang. Learn.* 44 283–305. 10.1111/j.1467-1770.1994.tb01103.x

[B65] Mackey A. Gass S. M. (2012). *Research Methods in Second Language Acquisition: A Practical Guide.* West Sussex: John Wiley & Sons, 10.1002/9781444347340

[B66] McHugh M. L. (2012). Lessons in biostatistics interrater reliability?: the kappa statistic. *Biochem. Medica* 22 276–282. PMC3900052 23092060

[B67] Mills N. Pajares F. Herron C. (2006). A reevaluation of the role of anxiety: self-efficacy, anxiety, and their relation to reading and listening proficiency. *Foreign Lang. Ann.* 39 276–295. 10.1111/j.1944-9720.2006.tb02266.x

[B68] Mills N. Pajares F. Herron C. (2007). Self-efficacy of college intermediate French students: relation to achievement and motivation. *Lang. Learn.* 57 417–442. 10.1111/j.1467-9922.2007.00421.x

[B69] Moghadam S. B. Ghanizadeh A. Akbari O. (2015). The effect of Bilingualism on the listening strategies and listening anxiety among Iranian Junior high school students. *J. Appl. Linguist. Lang. Res.* 2 236–248.

[B70] Mohammadi Golchi M. (2012). Listening anxiety and its relationship with listening strategy use and listening comprehension among Iranian IELTS learners. *Int. J. English Linguist* 2:115. 10.5539/ijel.v2n4p115

[B71] Moher D. Shamseer L. Clarke M. Ghersi D. Liberati^∧^ A. Petticrew M. (2015). Preferred reporting items for systematic review and meta-analysis protocols (PRISMA-P) 2015 statement. *Syst. Rev.* 4 1–9. 25554246 10.1186/2046-4053-4-1 PMC4320440

[B72] Movahed R. (2014). The effect of metacognitive strategy instruction on listening performance, Metaconitive awareness and listening anxiety of Beginner Iranian EFL Students. *Int. J. English Linguist.* 4 88–99. 10.5539/ijel.v4n2p88

[B73] Naghadeh M. A. Naghadeh N. Kasraey S. Maghdour H. (2014). The relationship between anxiety and Iranian EFL learners ’ narrative writing performance. *Int. Res. J. Manag. Sci.* 3 602–609.

[B74] Namaziandost E. Hafezian M. Shafiee S. (2018). Exploring the association among working memory, anxiety and Iranian EFL learners’ listening comprehension. *Asian-Pacific J. Second Foreign Lang. Educ* 3:20. 10.1186/s40862-018-0061-3

[B75] Niimoto S. (2021). Shadowing to alleviate listening anxiety and facilitate the development of bottom-up skills. *Stud. English Lang. Teach.* 44 101–109.

[B76] Noro T. (2006). Developing a construct model of” listening Stress”: a qualitative study of the affective domain of the listening process. *ARELE Annu. Rev. English Lang. Educ. Japan* 17 61–70.

[B77] Noro T. (2010). Debilitating effects of “listening stress”: focusing on the use of coping strategies. *ARELE Annu. Rev. English Lang. Educ. Jpn.* 21 201–210.

[B78] Nurkhamidah N. (2020). Exploring factors causing listening anxiety on generation Z students. *Acitya J. Teach. Educ.* 2 141–151.

[B79] Otair I. Abd Aziz N. H. (2017). Exploring the causes of listening comprehension anxiety from EFL Saudi learners’. *Perspectives Pilot Study. Adv. Lang. Lit. Stud.* 8:79. 10.7575/aiac.alls.v.8n.4p.79

[B80] Oxford R. (1990). *Language Learning Strategies: What Every Teacher Should Know.* Nashua, NH: Heinle & Heinle Publishers.

[B81] Oxford R. L. (1993). Research update on teaching L2 listening. *System* 21 205–211. 10.1016/0346-251X(93)90042-F

[B82] Pae T.-I. (2013). Skill-based L2 anxieties revisited: their intra-relations and the inter-relations with general foreign language anxiety. *Appl. Linguist.* 34 232–252.

[B83] Page M. J. McKenzie J. E. Bossuyt P. M. Boutron I. Hoffmann T. C. Mulrow C. D. (2021). The PRISMA 2020 statement: an updated guideline for reporting systematic reviews. *BMJ* 372:n71. 10.1136/bmj.n71 33782057 PMC8005924

[B84] Pan Y. E. (2016). Analysis of listening anxiety in EFL class. *Int. J. Stud. English Lang. Lit.* 4 12–16. 10.20431/2347-3134.0406002

[B85] Pekrun R. Goetz T. Frenzel A. C. Barchfeld P. (2011). Measuring emotions in students’ learning and performance. *Contemp. Educ. Psychol.* 36 36–48.

[B86] Plonsky L. (2013). Study quality in SLA. *Stud. Second Lang. Acquis.* 35 655–687. 10.1017/S0272263113000399

[B87] Plonsky L. (2015). *Advancing Quantitative Methods in Second Language Research. First Edit.* New York, NY: Routledge.

[B88] Plonsky L. Gass S. (2011). Quantitative research methods, study quality, and outcomes: the case of interaction research. *Lang. Learn.* 61 325–366. 10.1111/j.1467-9922.2011.00640.x

[B89] Polat M. Eristi B. (2019). The effects of authentic video materials on foreign language listening skill development and listening anxiety at different levels of english proficiency. *Int. J. Contemp. Educ. Res.* 6 135–154.

[B90] Rahimi M. Soleymani E. (2015). The impact of mobile learning on listening anxiety and listening comprehension. *English Lang. Teach.* 8 152–161.

[B91] Ranto Rozak R. Saleh M. Linggar Bharati D. A. Sutopo D. (2019). Reading while listening (RWL) in an extensive listening course to reduce student teachers’ foreign language listening anxiety (FLLA). *KnE Soc. Sci.* 3 349. 10.18502/kss.v3i10.3916

[B92] Renandya W. A. Farrell T. S. C. (2011). ‘Teacher, the tape is too fast!” extensive listening in ELT. *ELT J.* 65 52–59. 10.1093/elt/ccq015

[B93] Rezaabadi O. T. (2016). The relationships between social class, listening test anxiety and test scores. *Adv. Lang. Lit. Stud* 7 147–156. 10.7575/aiac.alls.v.7n.5p.147

[B94] Sarason I. G. (1984). Stress, anxiety, and cognitive interference: reactions to tests. *J. Pers. Soc. Psychol.* 46 929–938. 10.1037/0022-3514.46.4.929 6737201

[B95] Scovel T. (1978). The effect of affect on foreign language learning: a review of the anxiety research. *Lang. Learn.* 28 129–142. 10.1111/j.1467-1770.1978.tb00309.x

[B96] Serraj S. Noordin N. B. (2013). Relationship among Iranian EFL Students’ foreign language anxiety, foreign language listening anxiety and their listening comprehension. *English Lang. Teach.* 6 1–12.

[B97] Siddaway A. P. Wood A. M. Hedges L. V. (2018). How to do a systematic review: a best practice guide for conducting and reporting narrative reviews, meta-analyses, and meta-syntheses. *Annu. Rev. Psychol.* 70 747–770. 10.1146/annurev-psych-010418-102803 30089228

[B98] Spielberger C. H. A. D. (1971). Development of the Spanish edition of the state-trait anxiety inventory1. *Interam. J. Psychol* 5 145–158.

[B99] Tsai C.-C. (2013). The effects on listening strategies and listening anxiety by listening training program among EFL senior high school students in Taiwan. *Mod. J. Lang. Teach. Methods* 3 83–94.

[B100] Uchihara T. Webb S. Yanagisawa A. (2019). The effects of repetition on incidental vocabulary learning: a meta-analysis of correlational studies. *Lang. Learn.* 69 559–599. 10.1111/lang.12343

[B101] Vafaee P. Suzuki Y. (2019). The relative significance of syntactic knowledge and vocabulary knowledge in second language listening ability. *Stud. Second Lang. Acquis.* 42 383–410. 10.1017/S0272263119000676

[B102] Valizadeh M. R. Alavinia P. (2013). Listening comprehension performance viewed in the light of emotional intelligence and foreign language listening anxiety. *English Lang. Teach.* 6 11–26.

[B103] Vandergrift L. (2007). Recent developments in second and foreign language listening comprehension research. *Lang. Teach.* 40 191–210. 10.1017/S0261444807004338

[B104] Vandergrift L. Goh C. C. M. (2012). *Teaching and Learning Second Language Listening: Metacognition in Action.* Milton Park: Routledge, 10.4324/9780203843376

[B105] Vogely A. J. (1998). Listening comprehension anxiety: students’ reported sources and solutions. *Foreign Lang. Ann.* 31 67–80. 10.1111/j.1944-9720.1998.tb01333.x

[B106] Wang S. (2010). An experimental study of Chinese English major students’ listening anxiety of classroom learning activity at the university level. *J. Lang. Teach. Res.* 1 562–568. 10.4304/jltr.1.5.562-568

[B107] Wang S. (2016). Correlation between listening anxiety and listening strategies of Chinese postgraduate students of science and engineering: a case study at SUES. *Int. J. Learn. Dev.* 6 1–11. 10.5296/ijld.v6i4.10123

[B108] Wang S. Y. Cha K. W. (2019). Foreign language listening anxiety factors affecting listening performance of Chinese EFL learners. *J. Asia TEFL* 16 121–134.

[B109] Wheeless L. R. (1975). An investigation of receiver apprehension and social context dimensions of communication apprehension. *Speech Teach.* 24 261–268. 10.1080/03634527509378169

[B110] Xu F. (2011). Anxiety in EFL listening comprehension. *Theory Pract. Lang. Stud.* 1 1709–1717. 10.4304/tpls.1.12.1709-1717

[B111] Xu J. (2017). The mediating effect of listening metacognitive awareness between test-taking motivation and listening test score?: an expectancy-value theory approach. *Front. Psychol.* 8:2201. 10.3389/fpsyg.2017.02201 29312063 PMC5744478

[B112] Xu J. Huang Y.-T. (2018). The mediating effect of listening metacognitive awareness between listening test anxiety and listening test performance. *Asia Pacific Educ. Res.* 27 313–324.

[B113] Yamauchi Y. (2014a). Multifaceted images of Japanese EFL learners’ listening anxiety attributable to instructional factors. *Int. J. Curric. Dev. Pract.* 16 1–11. 10.18993/jcrdaen.16.1_1

[B114] Yamauchi Y. (2014b). Revised version of the foreign language listening anxiety scale?: precise description of subordinate concepts’ influence on learners. *ARELE Annu. Rev. English Lang. Educ. Jpn.* 25 143–158. 10.20581/arele.25.0_143

[B115] Yan J. X. Horwitz E. K. (2008). Learners’ perceptions of how anxiety interacts with personal and instructional factors to influence their achievement in english: a qualitative analysis of EFL learners in China. *Lang. Learn.* 58 151–183. 10.1111/j.1467-9922.2007.00437.x

[B116] Yanagisawa A. Webb S. (2021). To what extent does the involvement load hypothesis predict incidental L2 vocabulary learning? A meta-analysis. *Lang. Learn.* 71 487–536. 10.1111/lang.12444

[B117] Yang X. (2010). Intentional forgetting, anxiety, and EFL listening comprehension among Chinese college students. *Learn. Individ. Differ.* 20 177–187.

[B118] Yassin A. A. Razak N. A. (2017). Investigating the relationship between foreign language anxiety in the four skills and year of study among Yemeni University EFL learners. 3L Lang. *Linguist. Lit.* 23 147–159. 10.17576/3L-2017-2303-11

[B119] Young D. J. (1990). An investigation of students’ perspectives on anxiety and speaking. *Foreign Lang. Ann.* 23 539–553. 10.1111/j.1944-9720.1990.tb00424.x

[B120] Young D. J. (1991). Creating a low-anxiety classroom environment: what does language anxiety research suggest? *Mod. Lang. J.* 75 426–437. 10.1111/j.1540-4781.1991.tb05378.x

[B121] Zhai L. (2015). Influence of anxiety on English listening comprehension: an investigation based on the freshmen of English majors. *Stud. Lit. Lang.* 11 40–47. 10.3968/7952

[B122] Zhang X. (2013). Foreign language listening anxiety and listening performance: conceptualizations and causal relationships. *System* 41 164–177. 10.1016/j.system.2013.01.004

[B123] Zhang X. (2019). Foreign language anxiety and foreign language performance: a meta-analysis. *Mod. Lang. J.* 103 763–781. 10.1111/modl.12590

