# Supplementary material for: A Systematic Review of Foreign Language Listening Anxiety: Focus on the Theoretical Definitions and Measurements
Source: Front Psychol. 2022 Jun 23;13:859021. doi: 10.3389/fpsyg.2022.859021 (PMC9260422; doi:10.3389/fpsyg.2022.859021)
Supplement: Supplementary file 1 [file Data_Sheet_1.zip › Supplementary Material 2. Data extraction of the included studies.pdf]

Supplementary Material 2. Data extraction of the included studies.

Code	Study	Title	Sample	Major	Age	L1	L2	Orginal definitions	Coding of orginal definitions	Themes	Methods	Measurements	Coding of measurements	Analysis techniques	Factor analysis
1	Afshar & Hamzavi, 2014	The Relationship among Reflective Thinking, Listening Anxiety and Listening Comprehension of Iranian EFL Learners: Does Proficiency make a Difference?	223	unclear	mixed age	Iranian	English	MacIntyre (1995) states, L2 listeners are anxious about mis/non-understanding and also worry about embarrassing results.	psychological	Relation between FLLA and listening achievement	quantitative	Kim, 2000	psychological	Advanced multivariate statistics	/
2	Agudo, 2013	An Investigation into Spanish EFL Learners' Anxiety	217	no major	secondary school students	Spainish	English	no definition	no definition	Different levels of FLLA	quantitative	Horwitz et al., 1986	situation-specific	Descriptive analysis	/
3	Ali, 2017	English Language Anxiety: Development and Validation of a Brief Measure	362	unclear	university students	Egyptian	English	Vogely (1998) posits that listening comprehension anxiety can undermine speech production because, in order to interact verbally, the listener must first understand what is being said.	social	Measurement of FLLA	quantitative	Ali, 2017	unknown	Advanced multivariate statistics	/
4	Angellia & Listyani, 2019	Freshmens anxiety in an intensive listening class: A qualitative study	80	non-English major	university students	Indonesian	English	It is believed that English as a Foreign Language (EFL) listeners would likely to worry about misunderstanding or non-understanding, and they also fear for their embarrassing outcomes (MacIntyre, 1995 in Golchi, 2012).	social	Sources and/or effects of FLLA	qualitative	/	/	Descriptive analysis，Qualitative analysis	/
5	Atasheneh & Izadi, 2012	The Role of Teachers in Reducing/Increasing Listening Comprehension Test Anxiety: A Case of Iranian EFL Learners	60	English major	university students	Iranian	English	MacIntyre and Gardner (1994) define foreign language learning anxiety as " the feeling of tension and apprehension especially associated with second language context, including speaking, listening, reading and writing"	psychological	Relation between FLLA and listening achievement	quantitative	Horwitz et al., 1986	situation-specific	Conventional inferential statistics	/
6	Babakhouya & Elkhadiri,2019	An Investigation of the Relation between Neuroticism and English Language Listening Anxiety	328	non-English major	university students	Arabic	English	Listening was dealt with as a passive language skill that may be developed and mastered through classroom interaction, along with the belief that it is hard for language teachers to recognize learners who feel uncomfortable with listening ac- tivities and tasks, which is not the case with other language skills (Vogely, 1999; Bekleyen, 2009).	social	Relation between FLLA and psychological variables	quantitative	Kim, 2000	psychological	Conventional inferential statistics	/
7	Bang & Hiver, 2016	Investigating the Structural Relationships of Cognitive and Affective Domains for L2 Listening	300	no major	secondary school students	Korean	English	Anxiety can be broadly defined as a psychological state characterized by feelings of fear, tension or worry, and uneasiness (MacIntyre and Gregersen 2012). The unique characteristics of L2 listening, including one's inability to control the topic, speed, or volume of the speech, have the potential to create the experience of apprehension and helplessness in L2 learners relatively easily (Brunfaut and Révész 2015).	psychological	Relation between FLLA and listening achievement; Relation between FLLA and other afftctive variables	quantitative	Kim 2005	psychological	Advanced multivariate statistics	/
8	Bekleyen, 2009	Helping Teachers Become Better English Students: Causes, Effects, and Coping Strategies for Foreign Language Listening Anxiety	84	English major	university students	Turkish	English	(I) Foreign language listening anxiety (FLLA) is the type of anxiety experienced by language learners in situations that require listening.<br>(II) (anxiety) described by MacIntyre and Gardner (1994) as ''the feeling of tension and apprehension specifically associated with second language contexts, including speaking, listening and learning" (p. 284).	mixed (situation-specific + psychological)	Different levels of FLLA	mixed	Kim 2005	psychological	Conventional inferential statistics; Qualitative analysis	/
9	Berber & Gönen, 2017	How Do High and Low Anxious FL Listeners Employ FL Listening Comprehension Strategies? Exploring Student Perspectives	28	English major	university students	Turkish	English	While listening in the target language, feelings of anxiety may sabotage comprehension of the input and speech production which in turn affect interaction in a negative way (Vogely, 1998).	social	Relation between FLLA and other afftctive variables	quantitative	Kim, 2000	psychological	Descriptive analysis	/
10	Brunfaut & Révész, 2014	The Role of Task and Listener Characteristics in Second Language Listening	93	unclear	mixed age	Mixed	English	Listening anxiety is a type of situation-specific anxiety (MacIntyre & Gardner, 1991) that learners may uniquely experience when engaged in L2 listening.	situation-specific	Relation between FLLA and psychological variables	quantitative	Elkhafaifi, 2005	unknown	Conventional inferential statistics	/
11	Capan & Karaca, 2013	A Comparative Study of Listening Anxiety and Reading Anxiety	159	English major	university students	Turkish	English	Vogely (1998: 68) ng comprehension anxiety can undermine speech production because, in order to interact verbally, the listener must first understand what is being said.	social	Different levels of FLLA	quantitative	Kim, 2000	psychological	Conventional inferential statistics	/
12	Cebreros, 2003	Measuring language anxiety perceived by Spanish university students of English	33	English major	university students	Spanish	English	Listening anxiety reactions were not as frequent as those corresponding to speaking anxiety, since items 29 and 4 showed that only about 25% of the students felt restless when they didn't understand what the teacher said in the foreign language.	psychological	Different levels of FLLA	quantitative	Horwitz et al., 1986	situation-specific	Descriptive analysis	/
13	Chang & Read, 2008	Reducing Listening Test Anxiety through Various Forms of Listening Support	160	non-English major	university students	Chinese	English	Horwitz, Horwitz, and Cope (1986) propose that foreign language anxiety is a distinct variable composed of three components, the most relevant of which (for the present study) is test anxiety. Their conception received empirical support from the work of MacIntyre and Gardner (1989), who demonstrate through factor analysis that foreign language anxiety is separable from general anxiety, and that it is negatively correlated with achievement in the foreign language.	situation-specific	Different levels of FLLA	quantitative	Chang, 2008b	situation-specific; social	Conventional inferential statistics	/
14	Chang, 2008b	Sources of listening anxiety in learning English as a foreign language	160	non-English major	university students	Chinese	English	There are no tests which capture listeners' feelings in general and in testing situations. Therefore, a questionnaire was developed to identify college foreign language learners' listening anxiety in both general and test situations.	situation-specific	Measurement of FLLA	quantitative	Chang, 2008b	situation-specific; social	Advanced multivariate statistics	low confidence in comprehending spoken English/taking English listening courses as a requirement/worrying about test difficulty
15	Chang, 2008a	Listening Strategies of L2 Learners with Varied Test Tasks	22	non-English major	university students	Chinese	English	In general, language competence is undeniably an essential factor that affects anxiety; however, in a test situation, test task characteristics are also important variables that affect test-takers' performance (Bachman & Palmer, 1996). The characteristics of test tasks include previewing questions, multiple listening, sufficient background or linguistic knowledge, and being familiar with the test format. All these variables affect learners' listening anxiety to a certain extent (Chang, 2005b).	situation-specific	Relation between FLLA and other afftctive variables	quantitative	Chang, 2008b	situation-specific; social	Descriptive analysis	/
16	Chang, 2010	Second-language listening anxiety before and after a 1-yr. intervention in extensive listening compared with standard foreign language instruction	92	English major	university students	Chinese	English	Aneiro (1989) investigated Puerto Rican college students' listening apprehension and found that receiver apprehension was most affected by listening competence.	social	Relation between FLLA and instructional applications	quantitative	Chang, 2008b	situation-specific; social	Advanced multivariate statistics	/
17	Chen & Lin, 2014	A Study on the Relationship of English Listening Comprehension to Linguistic , Cognitive and Affective Variables among Taiwanese Elementary School Students	141	unclear	primary school students	Chinese	English	Kurita (2012) concluded that cognitive variables (e.g., L1 listening ability and metacognitive awareness), linguistic variables, (e.g., general L2 proficiency, L2 vocabulary knowledge, and phonological modification) and affective variables (e.g., listening anxiety and motivation) were found to contribute to L2 listening comprehension performance.	psychological	Relation between FLLA and psychological variables	quantitative	Elkhafaifi, 2005	unknown	Conventional inferential statistics	/
18	Cheng, 2017	Development and preliminary validation of four brief measures of L2 language-skill-specific anxiety	532	non-English major	university students	Chinese	English	no definition	no definition	Measurement of FLLA	quantitative	Cheng, 2017	psychological, physiological	Advanced multivariate statistics	cognitive/somatic/behavioral
19	Choi & Chon, 2014	Listener anxiety and listening strategies on multiple-choice items of EFL learners	121	unclear	secondary school students	Korean	English	While Field (2008) defines listener anxiety as the "fear that connected L2 speech is too difficult to make sense of" (p. 348)	psychological	Relation between FLLA and other afftctive variables; Different levels of FLLA, Measurement of FLLA	mixed	Choi & Chon, 2014	Fl listening ability; psychological, situation-specific	Conventional inferential statistics; Qualitative analysis	concern about insufficient prior knowledge/lack of self-confidence/testing anxiety
20	Chow et al., 2018	Anxiety in reading and listening English as a foreign language in Chinese undergraduate students	306	unclear	university students	Chinese	English	Listening anxiety could form under the false assumption that one must exhibit faultless comprehension and recognize all of the vocabulary (Scarcella & Oxford, 1992; Vogely, 1998).	social	Relation between FLLA and other afftctive variables	quantitative	Elkhafaifi, 2005	unknown	Advanced multivariate statistics	/
21	Elkhafaifi, 2005	Listening comprehension and anxiety in the Arabic language classroom	233	non-English major	mixed age	English	Arabic	no definition	no definition	Measurement of FLLA	quantitative	Elkhafaifi, 2005	unknown	Conventional inferential statistics	/
22	Fathi et al., 2020	The Effect of Listening Strategy Instruction on Second Language Listening Anxiety and Self-Efficacy of Iranian EFL Learners	52	English major	university students	Iranian	English	Listening anxiety pervasively exists in doing L2 listening tasks mainly because of the variables such as unintelligibility, perceived difficulty, unfamiliarity of tasks, and fear of failure in comprehension (Elkhafaifi, 2005).	psychological	Relation between FLLA and instructional applications	quantitative	Kim, 2000	psychological	Advanced multivariate statistics	/
23	Halat & Özbay, 2018	The Examination of Listening Anxiety Level of the Students Who Learn Turkish as a Foreign Language	187	unclear	mixed age	unclear	Turkish	According to Gardner and MacIntyre, foreign language anxiety is defined as a feeling of tension and concern that is directly related to education of foreign and second languages which includes learning, listening and speaking.	psychological	Different levels of FLLA	mixed	Kim, 2000	psychological	Conventional inferential statistics; Qualitative analysis	/
24	Hamid & Idrus, 2021	A Correlational Study of the English Listening and Speaking Anxiety in Rural Areas	311	unclear	secondary school students	Malay	English	This distinction is crucial as test anxiety manifests in all situation where a learner's knowledge and performance are measured, whereas Foreign Language Classroom Anxiety by Horwitz et al. (1986) is anxiety manifesting generally inside a second/foreign language classroom which is non-native to the learners.	situation-specific	Relation between FLLA and other anxieties	quantitative	Kimura, 2008	psychological	Conventional inferential statistics	/
25	Horwitz et al, 1986	Foreign Language Classroom Anxiety	225	unclear	university students	English	French, Spanish	We conceive foreign language anxiety as a distinct complex of self-perceptions, beliefs, feelings, and behaviors related to classroom language learning arising from the uniqueness of the language learning process.	situation-specific	Relation between FLLA and other anxieties	quantitative	Horwizt, 1986	situation-specific	Conventional inferential statistics	/
26	Hutapea et al., 2020	The Correlation between EFL ' s Students Listening Motivation with Listening Anxiety in Intermediate Listening Classes	60	English major	university students	Indonesian	English	In the listening process, listeners should focus on what they listen. It makes the students feel anxiety for what they have listened because they do not want to get the low score in listening class.	situation-specific	Relation between FLLA and other afftctive variables	quantitative	Kim, 2000	psychological	Conventional inferential statistics	/
27	Jee, 2018	Four skill-based foreign language anxieties: Learners of Korean in Australia	110	non-English major	university students	English	Korean	Horwitz et al. (1986) defined FLA as a situation-specific construct, which occurs only in certain situations, and distinguished from other types of anxiety such as trait anxiety and state anxiety (MacIntyre and Gardner 1989).	situation-specific	Relation between FLLA and other anxieties	mixed	Kim, 2000	psychological	Conventional inferential statistics; Qualitative analysis	/

28	Kaivanpanah et al., 2020	Examining the Effect of Listening Strategy Instruction on EFL Iraqi learners' Listening Anxiety	60	English major	university students	Arabic	English	(I) Psychologically, anxiety is as a personal feeling of unease or fear towards certain situations (MacIntyre & Gregersen, 2012), which may impede or interfere negatively with learners' input, processes, or performance in academic circumstances (Krashen, 1982).<br>(II) Drawing on these three conceptual anxiety dimensions, Horwitz et al. (1986) defined FL anxiety as a "distinct complex of self-perceptions, beliefs, feelings, and behaviors related to classroom language learning arising from the uniqueness of the language learning process" (p. 128).	mixed (psychological + situation-specific)	Relation between FLLA and instructional applications	quantitative	Elkhafaifi, 2005	unknown	Conventional inferential statistics	/
29	Kiliç & Uçkun, 2012	Listening text type as a variable affecting listening comprehension anxiety	130	non-English major	university students	Turkish	English	MacIntyre (1995a) also emphasized the FL students' worry about misunderstanding linguistic structures or inferring meaning from situational context because they make embarrassing mistakes in such activities.	social	Sources and/or effects of FLLA	quantitative	Kim, 2000	psychological	Conventional inferential statistics	/
30	Kim, 2000	Foreign language listening anxiety: a study of Korean students learning English	245	non-English major	university students	Korean	English	Communication apprehemsion (is) defined as "an individual's level of fear or anxiety associated with either real or anticipated communication with other person or persons" (McCroskey, 1970, p.269). Wheeless defined "receiver apprehension" as "the fear of misinterpreting, inadequately processing and/or not being able to adjuct psychologically to messages sent by others" (1975, p. 263).	social	Measurement of FLLA	quantitative	Kim, 2000	psychological	Advanced multivariate statistics	tension and worry over English listening/lack of confidence in listening
31	Kim, 2002	Affective reactions to foreign language listening retrospective interviews with Korean EFL students	20	non-English major	university students	Korean	English	Oxford (1993) regarded the learning goals and beliefs of listeners as a source of anxiety, mentioning that anxiety frequently occurs when students feel they cannot handle an L2 listening activity. For example, listeners tend to assume that they must understand every word they hear, even though they do not have to do so to understand what they hear in their native language.	social	Sources and/or effects of FLLA	qualitative	/	/	Qualitative analysis	/
32	Kim, 2011	Korean EFL Learners' Listening Anxiety, Listening Strategy Use, and Listening Proficiency	144	unclear	university students	Korean	English	MacIntyre and Gardner (1994), proposing a definition specific to language learning, referred language anxiety as the feeling of tension and apprehension specifically related to second language contexts, including speaking, listening, and learning.	psychological	Relation between FLLA and other affctive variables	quantitative	Kimura, 2008	psychological	Conventional inferential statistics	/
33	Kimura, 2008	Foreign Language Listening Anxiety: Its Dimensionality and Group Differences	452	non-English major	university students	Japanese	English	In this paper, one of the skill-based constructs, foreign language listening anxiety (FLLA), is investigated to explore the internal structure of this psychological construct using the statistical method of factor analysis	psychological	Measurement of FLLA	quantitative	Kim,2000	psychological	Advanced multivariate statistics	emotionality/worry/anticipatory fear
34	Kimura, 2011	A self-presentational presective of foreign language listening anxiety	1177	mixed	university students	Japanese	English	Wheeless (1975), in studying communication apprehension, stated that communicators can worry about both sending and receiving information and that they might represent different dimensions of communicative anxiety. Senders experience fear of social dissonance or exclusion when their communication is perceived as inefficient, meaningless, or inappropriate, whereas receivers arefearful of improperly perceiving messages and of responding inappropriately.	social	Measurement of FLLA	quantitative	Kimura, 2011	learner characteristics; sources of anxiety	Advanced multivariate statistics	self-focused apprehension/task-focused apprehension
35	Kimura, 2017	Foreign Language Listening Anxiety: A Self-Presentational View	1177	mixed	university students	Japanese	English	I propose that L2 listening anxiety is, at least in part, socially constructed. It is plausible that listeners' self-images as skillful L2 listeners are threatened because they worry about their ability to successfully comprehend aural input that they perceive as challenging.	social	Different levels of FLLA，Measurement of FLLA	quantitative	Kimura, 2011	learner characteristics; sources of anxiety	Advanced multivariate statistics	self-focused apprehension/task-focused apprehension
36	Ko, 2010	The effects of pedagogical agents on listening anxiety and listening comreohension in English as a foreign languge context	66	non-English major	university students	Korean	English	There is a particular paucity of research in listening anxiety (Elkhafaifi, 2005), defined as nervousness and fear of listening in a foreign language.	psychological	Relation between FLLA and instructional applications	quantitative	Elkhafaifi, 2005	unknown	Advanced multivariate statistics	/
37	Kutuk et al., 2019	Development and Validation of a New Multidimensional Language Class Anxiety Scale	323+701	unclear	university students	Turkish	English	The idea of developing distinct situation-specific measures assessing L2 anxiety was first implemented by Horwitz, Horwitz, and Cope (1986).Furthermore, we need to acknowledge the importance of the role of test anxiety in language classrooms.	situation-specific	Measurement of FLLA	quantitative	Kutuk et al., 2019	psychological, physiological	Advanced multivariate statistics	affective/cognitive/physiological
38	Lee, 2016	Computer-detected attention affects foreign language listening but not reading performance	252	English major	university students	Chinese	English	Situation-specific anxiety means the probability of becoming anxious in a certain situation (MacIntyre & Gardner, 1994), for instance, when using a foreign language, as shown by the foreign language reading anxiety scales (FLRAS) and foreign language listening anxiety scales (FLLAS).	situation-specific	Relation between FLLA and psychological variables	quantitative	Elkhafaifi, 2005	unknown	Conventional inferential statistics	/
39	Li, 2015	A Study of EFL Listening Anxiety in a Test Setting	102	English majors	university students	Chinese	English	Anxiety is a complex psychological construct consisting of many variables. It is difficult to collapse them all into a single concise definition. Linguists have come up defections from various aspects. (Spielberger, 1983; MacIntyre & Gardner, 1994; Madigan et al., 1996; Sellers, 2000).	psychological	Measurement of FLLA	mixed	Li, 2015	psychological, physiological	Conventional inferential statistics; Qualitative analysis	cognitive/affective/behavioral
40	Liu, 2016	Interrelations between Foreign Language Listening Anxiety and Strategy Use and Their Predicting Effects on Test Performance of High- and Low-Proficient Chinese University	1160	non-English major	university students	Chinese	English	(I) Defined as the ''apprehension experienced when a situation requires the use of a second language with which the individual is not fully proficient'' (MacIntyre and Gardner 1994, p. 5), FLA has been found to be existent in all aspects of SL/FL learning such as listening, speaking, reading and writing and mainly debilitates the learning of a SL/FL (Ewald 2007; Dewaele and Al-Saraj 2015; Dewaele and Tsui 2013; Horwitz et al. 1986; MacIntyre and Gardner 1991, 1994; Marcos-Llina´s and Garau 2009; Tallon 2009).<br>(II) Listening anxiety has been found to exist in SL/FL listening-related tasks due to such factors as incomprehensibility, task difficulty, task unfa-miliarity, and fear of embarrassing outcomes (Elkhafaifi 2005; Samaneh and Noordin 2013).	mixed (psychological + situation-specific)	Relation between FLLA and other affctive variables	quantitative	Elkhafaifi, 2005	psychological; learner characteristics; FL listening ability	Conventional inferential statistics	/
41	MacIntyre & Gardner, 1994	The Subtle Effects of Language Anxiety on Cognitive Processing in the Second Language	97	unclear	university students	English	French	Language anxiety can be defined as the feeling oftension and apprehension specifically associated with second language contexts, including speaking, listening, and learning.	psychological	Relation between FLLA and psychological variables	quantitative	MacIntyre & Gardner, 1994	situation-specific	Conventional inferential statistics	/
42	MacIntyre, 1995	How Does Anxiety Affect Second Language Learning? A Reply to Sparks and Ganschow	/	no major	/	English	Unclear	(I) Social anxiety is defined by "(1) feelings of tension and discomfort, (2) negative self- evaluations, and (3) a tendency to withdraw in the presence ofothers" (Schwarzer, 1986, p. 1).<br>(II) The cognitive and affective components of anxiety were identified by Liebert and Morris (1967) as "worry" and "emotionality" respec- tively. Sarason (1986) defined worry as ". . . distressing preoccupations and concerns about impending events" (p. 21). This preoccupation often takes the form of self-related cognition, which is seldom beneficial for task performance.<br>(III) For some students, this is a frequent course of events, and anxiety becomes reliably associated with any situation involving the second language.	mixed (social + psychological + situation-specific)	Sources and/or effects of FLLA	qualitative	/	/	Qualitative analysis	/
43	Mills et al., 2006	A reevaluation of the role of anxiety: Self-efficacy, anxiety, and their relation to reading and listening proficiency	95	non-English major	university students	English and others	French	Bandura (1997) defined anxiety as "a state of anticipatory apprehension over possible deleterious happenings" (p. 137)	psychological	Relation between FLLA and other affctive variables; Relation between FLLA and listening achievement	quantitative	Mills et al., 2006	unknown	Advanced multivariate statistics	/
44	Mills et al., 2007	Self-Efficacy of College Intermediate French Students: Relation to Achievement and Motivation	303	non-English major	university students	English and others	French	French anxiety in reading and listening is the state ofanticipatory apprehension related to listening and reading in French. Bandura (1997) defined anxiety as "a state of anticipatory apprehension over possible deleterious happenings" (p. 137). Individuals experiencing anxiety embody apprehension and avoidant behavior that often interfere with performance in everyday life as well as in academic situations.	psychological	Relation between FLLA and other affctive variables; Relation between FLLA and listening achievement	quantitative	Mills et al., 2006	unknown	Conventional inferential statistics	/
45	Moghadam et al., 2015	The Effect of Bilingualism on the Listening Strategies and Listening Anxiety among Iranian Junior High School Students	200	no major	secordnary school students	Persian, Kurdish, Turkish, Turkemen	English	Foreign language listening anxiety was seen as a distinct type of situation-specific anxiety.	situation-specific	Relation between FLLA and other affctive variables	quantitative	Elkhafaifi, 2005	unknown	Conventional inferential statistics	/
46	Mohammadi Golchi, 2012	Listening Anxiety and Its Relationship with Listening Strategy Use and Listening Comprehension among Iranian IELTS Learners	63	no major	secordnary school students	Persian	English	(I) MacIntyre (1995) believed that listeners in L2 worry about mis-understanding or non-understanding, and they fear embarrassing outcomes.<br>(II) During listening process, different factors may cause uneasiness and tension for language learners and result in poor listening.	mixed (social + psychological)	Relation between FLLA and other affctive variables; Relation between FLLA and listening achievement	quantitative	Kim, 2000	psychological	Conventional inferential statistics	/
47	Movahed, 2014	The Effect of Metacognitive Strategy Instruction on Listening Performance, Metaconitive awareness and Listening anxiety of Beginner Iranian EFL Students	55	English major	university students	Iranian	English	Bekleyen (2009) defines foreign language listening anxiety (FLLA) as a kind of anxiety aroused in situations which need listening.	situation-specific	Relation between FLLA and instructional applications	quantitative	Kim, 2000	psychological	Conventional inferential statistics	/
48	Namaziandost et al., 2018	Exploring the association among working memory, anxiety and Iranian EFL learners' listening comprehension	60	unclear	university students	Persian	English	(I) MacIntyre (1995) states, L2 listeners are anxious about mis/non-understanding and also worry about embarrassing results.<br>(II) As an affective factor which might be individually based, test-taking anxiety has recently been studied in different contexts.	mixed (social + situation-specific)	Relation between FLLA and psychological variables	quantitative	Kim, 2000	psychological	Conventional inferential statistics	/
49	Niimoto, 2021	Shadowing to Alleviate Listening Anxiety and Facilitate the Development of Bottom-up Skills	116	non-English major	university students	Japanese	English	Foreign language anxiety is defined as "the feeling of tension and apprehension specifically associated with second-language contexts, including speaking, listening and learning" (MacIntyre, & Gardner, 1994, p. 284). Listening anxiety over a foreign language is one such a feeling that learners experience while listening (Yamauchi, 2014b).	psychological	Relation between FLLA and other affctive variables	quantitative	Yamauchi, 2014c	sources of anxiety	Conventional inferential statistics	/

50	Noro, 2006	Developing a construct model of" listening Stress": A qualitative study of the affective domain of the listening process	22	unclear	university students	Japanese	English	Noro (2005a) introduced the psychological construct of listening stress as an alternative to listening anxiety in the affective domain of the listenjng process. Listening stress was defined as "psychological inhibition debilitating listening comprehension which L2/FL learners experience in the face of listening tasks that they perceive to be too difficult" (Noro, 2005b, p. 138).	psychological	Sources and/or effects of FLLA	qualitative	/	/	Qualitative analysis，Descriptive analysis	/
51	Noro, 2010	Debilitating Effects of "Listening Stress": Focusing on the Use of Coping Strategies	7	unclear	university students	Japanese	English	The conceptualizatien of "listening stress" was based on Lazarus and Folkman's (1984) psychological stress theory, defining it as "psychological inhibition debilitating listening comprehension which L2/FL learners experience in the face of listening tasks that they perceive to be too difficult" (Noro, 2005b, p. 138).	psychological	Relation between FLLA and other afftctive variables	quantitative	Maclntyre and Gardner, 1994	situation-specific	Descriptive analysis	/
52	Nurkhamidah, 2020	Exploring Factors Causing Listening Anxiety On Generation Z Students	15	English	university students	Indonesian	English	Listener not only receive the information from speakers, but they must process the meaning of the speakers' utterances. Comprehend people's utterance is not easy. Students in university find many problems in listening comprehension (Hamouda ,2013). This difficulty and complexity cause anxiety among second or foreign language learners (Graham, 2006).	social	Sources and/or effects of FLLA	qualitative	/	/	Qualitative analysis	/
53	Otair & Abd Aziz, 2017	Exploring the Causes of Listening Comprehension Anxiety from EFL Saudi Learners' Perspectives: A Pilot Study	2	non-English major	university students	Arabic	English	Language anxiety is defined as "the feeling of tension and apprehension, spe- cifically associated with second language contexts, including speaking, listening, and learning" (Maclntyre and Gardner, 1994, p.24).	psychological	Sources and/or effects of FLLA	qualitative	/	/	Qualitative analysis	/
54	Pae, 2013	Skill-Based L2 Anxieties Revisited: Their Intra-Relations and the Inter-Relations with General Foreign Language Anxiety	229	mixed	university students	Korean	English	It has been suggested that anxiety interferes with FL learning (Horwitz et al. 1986; MacIntyre and Gardner 1989; Price 1991; Saito and Samimy 1996), and anxious FL learners tend to show physiological as well as psycholinguistic symptoms.	psychological	Relation between FLLA and other anxieties	quantitative	Kim, 2000	psychological	Advanced multivariate statistics	/
55	Pan, 2016	Analysis of Listening Anxiety in EFL Class	/	no major	/	Chinese	English	Anxiety is defined as "the subjective feeling of tension, apprehension, nervousness, and worry associated with an arousal of the autonomic nervous system" (Spielberger, 1983, p. 1), in the eyes of Arnold and Brown (1999, p. 8), "is associated with negative feelings such as uneasiness, frustration, self-doubt, apprehension and tension."	psychological	Sources and/or effects of FLLA	qualitative	/	/	Qualitative analysis	/
56	Polat & Eristi, 2019	The Effects of Authentic Video Materials on Foreign Language Listening Skill Development and Listening Anxiety at Different Levels of English Proficiency	100	non-English major	university students	Turkish	English	foreign language listening anxiety can be defined as "feelings of apprehension, restlessness, tension, uneasiness and fear, experienced by language learners, stemming from actions required before and during the listening activity as well as other various stimuli" (Polat & Erişti, 2018)	psychological	Sources and/or effects of FLLA; Relation between FLLA and other anxieties，Measurement of FLLA	quantitative	Polat & Erişti, 2018	sources of anxiety	Conventional inferential statistics	individual and environmental elements/the control of listening sources/ascribed meaning to listening activities
57	Rahimi & Soleymani, 2015	The Impact of Mobile Learning on Listening Anxiety and Listening Comprehension	50	unclear	unclear	Persian	English	This type of anxiety which is associated with listening tasks is called listening anxiety.	situation-specific	Relation between FLLA and instructional applications	quantitative	Kimura, 2008	psychological	Advanced multivariate statistics	/
58	Ranto Rozak et al., 2019	Reading While Listening (RWL) in an Extensive Listening Course to Reduce Student Teachers' Foreign Language Listening Anxiety (FLLA)	57	English major	university students	Indonesian	English	It was identified that the characteristics of spoken texts such as the main ideas missing, disability to catch the keywords, the speed rate, and little time to process the aural input are the main variables of their FLLA (Chang, 2010).	social	Different levels of FLLA， Sources and/or effects of FLLA	quantitative	Kim, 2000	psychological	Conventional inferential statistics	/
59	Rezaabadi, 2016	The Relationships between Social Class, Listening Test Anxiety and Test Scores	78	non-English major	unclear	Iranian	English	According to MacIntyre and Gardner (1994), students suffering from FL anxiety are tense and apprehensive, especially when participating in listening and speaking activities in the second language. Students	psychological	Different levels of FLLA	quantitative	Elkhafaifi, 2005	unknown	Conventional inferential statistics	/
60	Serraj & Noordin, 2013	Relationship among Iranian EFL Students' Foreign Language Anxiety, Foreign Language Listening Anxiety and Their Listening Comprehension	210	non-English major	unclear	Farsi	English	MacIntyre (1995) explained the reason for such an anxiety is that learners often worry about misunderstanding what they listen to and the fear of being embarrassed by interpreting the message wrongly (Chastain, 1979).	social	Relation between FLLA and listening achievement	quantitative	Kim, 2000	psychological	Conventional inferential statistics	/
61	Tsai, 2013	The effects on listening strategies and listening anxiety by listening training program among EFL senior high school students in Taiwan	124	no major	secondary school students	Chinese	English	Vogely (1998) showed many potential sources of listening comprehension anxiety based on students' reports: input that is not clear or that is given too fast, and students' belief that they have to make sense of every single word.	social	Relation between FLLA and other afftctive variables	quantitative	Kim, 2000	psychological	Conventional inferential statistics	/
62	Vafaee & Suzuki, 2019	The relative significance of syntactic knowledge and vocabulary knowledge in second language listening ability	263	unclear	university students	Persian	English	(I) L2 anxiety is defined as the latter, situation-specific anxiety, meaning " the feeling of tension and apprehension specifically associated with second language context, including speaking, listening and learning" (MacIntyre & Gardner, 1994, p. 284).<br>(II) According to Scarcella and Oxford (1992), listening L2 anxiety usually occurs when learners feel they are faced with a difficult and unfamiliar L2 listening task. L2 listening anxiety increases when listeners are under the false impression that to complete a listening task, usually in a testing situation, they must understand every single word they hear (Vogely, 1998).	mixed (psychologial + situation-specific)	Sources and/or effects of FLLA; Relation between FLLA and listening achievement	quantitative	Elkhafaifi, 2005	unknown	Advanced multivariate statistics	/
63	Valizadeh & Alavinia, 2013	Listening Comprehension Performance Viewed in the Light of Emotional Intelligence and Foreign Language Listening Anxiety	160	English major	university students	Azeri Turkish, Kurdish, and Farsi	English	MacIntyre and Gardner (1994) delineate foreign language learning anxiety as "the feeling of tension and apprehension especially associated with second language context, including speaking, listening, reading and writing" (p. 288).	psychological	Relation between FLLA and psychological variables; Relation between FLLA and listening achievement	quantitative	Elkhafaifi, 2005	unknown	Conventional inferential statistics	/
64	Vogely， 1998	Listening comprehension anxiety: Students' reported sources and solutions	140	unclear	university students	English	Spanish	According to Scarcella and Oxford (1992), listening anxiety occurs when students feel they are faced with a task that is too difficult or unfamiliar to them. This anxiety is exacerbated if the listeners are under the false impression that they must understand every word they hear.	social	Sources and/or effects of FLLA	qualitative	/	/	Descriptive analysis, Qualitative analysis	/
65	Wang & Cha, 2019	Foreign Language Listening Anxiety Factors Affecting Listening Performance of Chinese EFL Learners	78	English major	university students	Chinese	English	FLLA is conceptualized as situation-specific and refers to the tendency that FL listeners become anxious in listening-related tasks (Liu, 2016; Zhang, 2013).	situation-specific	Relation between FLLA and listening achievement，Measurement of FLLA	quantitative	Wang & Cha, 2019	psychological; learner characteristics; FL listening ability	Conventional inferential statistics	listening-anxiety/self-belief /decoding-skills
66	Wang, 2010	An Experimental Study of Chinese English Major Students' Listening Anxiety of Classroom Learning Activity at the University Level	125	English major	university students	Chinese	English	(I) anxiety is an affective factor in listening comprehension;<br>(II) FLLA plays a very important role because the anticipation of foreign language use in receiving information can provoke anxiety.	mixed (psychological + social)	Relation between FLLA and listening achievement， Measurement of FLLA	quantitative	Wang, 2010	psychological, FL listening ability	Conventional inferential statistics	tension and worry over English listening/lack of confidence in English listening/concern about insufficient prior knowledge
67	Wang, 2016	Correlation between Listening Anxiety and Listening Strategies of Chinese Postgraduate Students of Science and Engineering: A Case Study at SUES	182	non-English major	university students	Chinese	English	anxiety is an important affective factor and should not be overlooked, because the anticipation of foreign language use in receiving information can provoke anxiety, especially anxious students might misunderstand linguistic structures or infer meaning from context for fear of making mistakes and losing face.	social	Relation between FLLA and other afftctive variables, Relation between FLLA and listening achievement	quantitative	Wang, 2010	psychological, FL listening ability	Conventional inferential statistics	/
68	Xu & Huang, 2018	The Mediating Effect of Listening Metacognitive Awareness between Listening Test Anxiety and Listening Test Performance	402	unclear	university students	Chinese	English	L2 listening comprehension can be broken by two components: foreign language listening anxiety (shortened as listening anxiety thereafter) and test anxiety (Vandergrift 2015)	situation-specific	Relation between FLLA and other afftctive variables; Relation between FLLA and listening achievement	quantitative	Elkhafaifi, 2005	psychological; learner characteristics; FL listening ability	Advanced multivariate statistics	/
69	Xu, 2011	Anxiety in EFL listening comprehension	140	non-English majors	university students	Chinese	English	The anxiety that arises during the listening process often springs form what Joiner (1986) calls a negative "listening self-concept," that is, a low level of self-confidence in the area of listening.	social	Sources and/or effects of FLLA	qualitative	/	/	Qualitative analysis	/
70	Xu, 2017	The Mediating Effect of Listening Metacognitive Awareness between Test-Taking Motivation and Listening Test Score : An Expectancy-Value Theory Approach	560	unclear	university students	Chinese	English	listening anxiety, referring to the upset or nervousness caused by the listening comprehension	psychological	Relation between FLLA and other afftctive variables	quantitative	Elkhafaifi, 2005	psychological; learner characteristics; FL listening ability	Advanced multivariate statistics	/
71	Yamauchi, 2014a	Multifaceted Images of Japanese EFL Learners' Listening Anxiety Attributable to Instructional Factors	142	non-English major	university students	Japanese	English	While listening, most learners worry about the nature of the input (Vogely, 1998).	psychological	Relation between FLLA and listening achievement	quantitative	Elkhafaifi, 2005	unknown	Advanced multivariate statistics	/
72	Yamauchi, 2014b	Revised Version of the Foreign Language Listening Anxiety Scale : Precise Description of Subordinate Concepts' Influence on Learners	996	non-English major	university students	Japanese	English	Foreign language anxiety is defined as "feeling of tension and apprehension specifically associated with second-1anguage contexts, including speaking, listening, and learning" (Macintyre & Gardner, 1994, p. 284). Foreign language listening anxiety, which this study addresses, is such a feeling that learners experience while listening.	psychological	Measurement of FLLA	quantitative	Yamauchi, 2014b	sources of anxiety, psychological	Advanced multivariate statistics	factors other than the material/factors related to the material itself/factors related to listeners' cognitive processes
73	Yang, 2010	Intentional Forgetting, Anxiety, and EFL Listening Comprehension among Chinese College Students	150+ 181	non-English major	university students	Chinese	English	The inclusion of somatic anxiety was based on the conceptualization of anxiety from Craft, Magyar, Becker and Feltz (2003), and it refers to one's perception of the physiological effects of the anxiety experience, as reflected in increased autonomic arousal and unpleasant feeling states such as nervousness and tension.	psychological	Relation between FLLA and listening achievement; Relation between FLLA and psychological variables， Measurement of FLLA	quantitative	Yang, 2010	physiological, learner characteristics	Conventional inferential statistics	somatic anxiety/self-efficacy
74	Yassin & Razak, 2017	Investigating the relationship between foreign language anxiety in the four skills and year of study among Yemeni University EFL learners	155	English major	university students	Arabic	English	FLCAS are speaking anxiety which includes two factors, namely communication apprehension and fear of negative evaluation, and listening anxiety which includes communication apprehension.	social	Relation between FLLA and listening achievement	quantitative	Horwitz et al., 1986	situation-specific	Conventional inferential statistics	/
75	Zhai, 2015	Influence of anxiety on English listening comprehension: An investigation based on the freshmen of English majors	82	English major	university students	Chinese	English	In the 1970s, some researchers began to realize and acknowledge the existence of listening anxiety in foreign language learning. Wheels (1975) described listening anxiety as "receiver's apprehension— the fear of misinterpreting, inadequately processing or not being able to adjust psychologically to messages sent by others."	social	Sources and/or effects of FLLA; Relation between FLLA and listening achievement	mixed	Kim, 2000	psychological	Conventional inferential statistics; Qualitative analysis	/
76	Zhang, 2013	Foreign language listening anxiety and listening performance: Conceptualizations and causal relationships	300	English major	university students	Chinese	English	FL listening anxiety refers to FL anxiety that is associated specifically with FL listening situations.	situation-specific	Measurement of FLLA; Relation between FLLA and listening achievement	quantitative	Zhang, 2013	psychological; learner characteristics; FL listening ability	Advanced multivariate statistics	listening anxiety/self-belief/FL listening decoding skills
