# Supplementary material for: A Systematic Review of Foreign Language Listening Anxiety: Focus on the Theoretical Definitions and Measurements
Source: Front Psychol. 2022 Jun 23;13:859021. doi: 10.3389/fpsyg.2022.859021 (PMC9260422; doi:10.3389/fpsyg.2022.859021)
Supplement: Supplementary file 1 [file Data_Sheet_1.zip › Supplementary Material 3. The measurements of FL listening anxiety.pdf]

Supplementary Material 3. The measurements of FL listening anxiety.

Studies	Measurements
Ali, 2017	Ali, 2017
Chang & Read, 2008<br>Chang, 2008b<br>Chang, 2008a<br>Chang, 2010	Chang, 2008b
Cheng, 2017	Cheng, 2017
Choi & Chon, 2014	Choi & Chon, 2014
Brunfaut & Révész, 2014<br>Chen & Lin, 2014<br>Chow et al., 2018<br>Elkhafaifi, 2005<br>Kaivanpanah et al., 2020<br>Ko, 2010<br>Lee, 2016<br>Moghadam et al., 2015<br>Rezaabadi, 2016<br>Vafaee & Suzuki, 2019<br>Valizadeh & Alavinia, 2013<br>Xu & Huang, 2018<br>Xu, 2017<br>Yamauchi, 2014a<br>Liu, 2016	Elkhafaifi, 2005
Agudo, 2013<br>Atasheneh & Izadi, 2012<br>Cebreros, 2003<br>Yassin & Razak, 2017<br>Horwitz et al, 1986	Horwitz et al., 1986
Bang & Hiver, 2016<br>Bekleyen, 2009	Kim 2005
Afshar & Hamzavi, 2014<br>Babakhouya & Elkhadiri,2019<br>Berber & Gönen, 2017<br>Capan & Karaca, 2013<br>Fathi et al., 2020<br>Halat & Özbay, 2018<br>Hutapea et al., 2020<br>Jee, 2018<br>Kiliç & Uçkun, 2012<br>Kim, 2000<br>Mohammadi Golchi, 2012<br>Movahed, 2014	Kim, 2000

Namaziandost et al., 2018	
Pae, 2013	
Ranto Rozak et al., 2019	
Serraj & Noordin, 2013	
Tsai, 2013	
Zhai, 2015	
Kimura, 2008	
Hamid & Idrus, 2021	
Kim, 2011	Kimura, 2008
Rahimi & Soleymani, 2015	
Kimura, 2011	Kimura, 2011
Kimura, 2017	
Kutuk et al., 2019	Kutuk et al., 2019
Li, 2015	Li, 2015
MacIntyre & Gardner, 1994	MacIntyre & Gardner, 1994
Noro, 2010	
Mills et al., 2006	Mills et al., 2006
Mills et al., 2007	
Polat & Eristi, 2019	Polat & Erişti, 2018
Wang & Cha, 2019	Wang & Cha, 2019
Wang, 2010	Wang, 2010
Wang, 2016	
Yamauchi, 2014b	Yamauchi, 2014b
Niimoto, 2021	
Yang, 2010	Yang, 2010
Zhang, 2013	Zhang, 2013
