# Supplementary material for: A Systematic Review of Foreign Language Listening Anxiety: Focus on the Theoretical Definitions and Measurements
Source: Front Psychol. 2022 Jun 23;13:859021. doi: 10.3389/fpsyg.2022.859021 (PMC9260422; doi:10.3389/fpsyg.2022.859021)
Supplement: Supplementary file 1 [file Data_Sheet_1.zip › Supplementary Material 1. The original definitions of the included studies.PDF]

Supplementary Material 1. The original definitions of the included studies.

Studies	Original definitions
Afshar & Hamzavi, 2014	MacIntyre (1995) states, L2 listeners are anxious about mis/non-understanding and also worry about embarrassing results.
Agudo, 2013	no definition
Ali, 2017	Vogely (1998) posits that listening comprehension anxiety can undermine speech production because, in order to interact verbally, the listener must first understand what is being said.
Angellia & Listyani, 2019	It is believed that English as a Foreign Language (EFL) listeners would likely to worry about misunderstanding or non-understanding, and they also fear for their embarrassing outcomes (MacIntyre, 1995 in Golchi, 2012).
Atasheneh & Izadi, 2012	MacIntyre and Gardner (1994) define foreign language learning anxiety as "the feeling of tension and apprehension especially associated with second language context, including speaking, listening, reading and writing".
Babakhouya & Elkhadiri,2019	Listening was dealt with as a passive language skill that may be developed and mastered through classroom interaction, along with the belief that it is hard for language teachers to recognize learners who feel uncomfortable with listening activities and tasks, which is not the case with other language skills (Vogely, 1999; Bekleyen, 2009).
Bang & Hiver, 2016	Anxiety can be broadly defined as a psychological state characterized by feelings of fear, tension or worry, and uneasiness (MacIntyre and Gregersen 2012). The unique characteristics of L2 listening, including one's inability to control the topic, speed, or volume of the speech, have the potential to create the experience of apprehension and helplessness in L2 learners relatively easily (Brunfaut and Révész 2015).
Bekleyen, 2009	(I) Foreign language listening anxiety (FLLA) is the type of anxiety experienced by language learners in situations that require listening. (II) (Anxiety) described by MacIntyre and Gardner (1994) as "the feeling of tension and apprehension specifically associated with second language contexts, including speaking, listening and learning" (p. 284).
Berber & Gönen, 2017	While listening in the target language, feelings of anxiety may sabotage comprehension of the input and speech production which in turn affect interaction in a negative way (Vogely, 1998).
Brunfaut & Révész, 2014	Listening anxiety is a type of situation-specific anxiety (MacIntyre & Gardner, 1991) that learners may uniquely experience when engaged in L2 listening.
Capan & Karaca, 2013	Vogely (1998: 68) posits that "listening comprehension anxiety can undermine speech production because, in order to interact verbally, the listener must first understand what is being said."
Cebreros, 2003	Listening anxiety reactions were not as frequent as those corresponding to speaking anxiety, since items 29 and 4 showed that only about 25% of the students felt restless when they didn't understand what the teacher said in the foreign language.
Chang & Read, 2008	Horwitz, Horwitz, and Cope (1986) propose that foreign language anxiety is a distinct variable composed of three components, the most relevant of which

	(for the present study) is test anxiety. Their conception received empirical support from the work of MacIntyre and Gardner (1989), who demonstrate through factor analysis that foreign language anxiety is separable from general anxiety, and that it is negatively correlated with achievement in the foreign language.
Chang, 2008b	There are no tests which capture listeners' feelings in general and in testing situations. Therefore, a questionnaire was developed to identify college foreign language learners' listening anxiety in both general and test situations.
Chang, 2008a	In general, language competence is undeniably an essential factor that affects anxiety; however, in a test situation, test task characteristics are also important variables that affect test-takers' performance (Bachman & Palmer, 1996). The characteristics of test tasks include previewing questions, multiple listening, sufficient background or linguistic knowledge, and being familiar with the test format. All these variables affect learners' listening anxiety to a certain extent (Chang, 2005b).
Chang, 2010	Aneiro (1989) investigated Puerto Rican college students' listening apprehension and found that receiver apprehension was most affected by listening competence
Chen & Lin, 2014	Kurita (2012) concluded that cognitive variables (e.g., L1 listening ability and metacognitive awareness), linguistic variables, (e.g., general L2 proficiency, L2 vocabulary knowledge, and phonological modification) and affective variables (e.g., listening anxiety and motivation) were found to contribute to L2 listening comprehension performance.
Cheng, 2017	no definition
Choi & Chon, 2014	While Field (2008) defines listener anxiety as the "fear that connected L2 speech is too difficult to make sense of" (p. 348).
Chow et al., 2018	Listening anxiety could form under the false assumption that one must exhibit faultless comprehension and recognize all of the vocabulary (Scarcella & Oxford, 1992; Vogely, 1998).
Elkhafaifi, 2005	no definition
Fathi et al., 2020	Listening anxiety pervasively exists in doing L2 listening tasks mainly because of the variables such as unintelligibility, perceived difficulty, unfamiliarity of tasks, and fear of failure in comprehension (Elkhafaifi, 2005).
Halat & Özbay, 2018	According to Gardner and MacIntyre, foreign language anxiety is defined as a feeling of tension and concern that is directly related to education of foreign and second languages which includes learning, listening and speaking.
Hamid & Idrus, 2021	This distinction is crucial as test anxiety manifests in all situation where a learner's knowledge and performance are measured, whereas Foreign Language Classroom Anxiety by Horwitz et al. (1986) is anxiety manifesting generally inside a second/foreign language classroom which is non- native to the learners.
Horwitz et al, 1986	We conceive foreign language anxiety as a distinct complex of self-perceptions, beliefs, feelings, and behaviors related to classroom language

	learning arising from the uniqueness of the language learning process.
Hutapea et al., 2020	In the listening process, listeners should focus on what they listen. It makes the students feel anxiety for what they have listened because they do not want to get the low score in listening class.
Jee, 2018	Horwitz et al. (1986) defined FLA as a situation-specific construct, which occurs only in certain situations, and distinguished from other types of anxiety such as trait anxiety and state anxiety (MacIntyre and Gardner 1989)
Kaivanpanah et al., 2020	(I) Psychologically, anxiety is as a personal feeling of unease or fear towards certain situations (MacIntyre & Gregersen, 2012), which may impede or interfere negatively with learners' input, processes, or performance in academic circumstances (Krashen, 1982).<br>(II) Drawing on these three conceptual anxiety dimensions, Horwitz et al. (1986) defined FL anxiety as a "distinct complex of self-perceptions, beliefs, feelings, and behaviors related to classroom language learning arising from the uniqueness of the language learning process" (p. 128).
Kiliç & Uçkun, 2012	MacIntyre (1995a) also emphasized the FL students' worry about misunderstanding linguistic structures or inferring meaning from situational context because they make embarrassing mistakes in such activities.
Kim, 2000	Communication apprehension (is) defined as "an individual's level of fear or anxiety associated with either real or anticipated communication with other person or persons" (McCroskey, 1970, p.269). Wheeless defined "receiver apprehension" as "the fear of misinterpreting, inadequately processing and/or not being able to adjuct psychologically to messages sent by others" (1975, p. 263).
Kim, 2002	Oxford (1993) regarded the learning goals and beliefs of listeners as a source of anxiety, mentioning that anxiety frequently occurs when students feel they cannot handle an L2 listening activity. For example, listeners tend to assume that they must understand every word they hear, even though they do not have to do so to understand what they hear in their native language.
Kim, 2011	MacIntyre and Gardner (1994), proposing a definition specific to language learning, referred language anxiety as the feeling of tension and apprehension specifically related to second language contexts, including speaking, listening, and learning.
Kimura, 2008	In this paper, one of the skill-based constructs, foreign language listening anxiety (FLLA), is investigated to explore the internal structure of this psychological construct using the statistical method of factor analysis
Kimura, 2011	Wheeless (1975), in studying communication apprehension, stated that communicators can worry about both sending and receiving information and that they might represent different dimensions of communicative anxiety. Senders experience fear of social dissonance or exclusion when their communication is perceived as inefficient, meaningless, or inappropriate, whereas receivers are fearful of improperly perceiving messages and of responding inappropriately.
Kimura, 2017	I propose that L2 listening anxiety is, at least in part, socially constructed. It is

	plausible that listeners' self-images as skillful L2 listeners are threatened because they worry about their ability to successfully comprehend aural input that they perceive as challenging.
Ko, 2010	There is a particular paucity of research in listening anxiety (Elkhafaifi, 2005), defined as nervousness and fear of listening in a foreign language.
Kutuk et al., 2019	The idea of developing distinct situation-specific measures assessing L2 anxiety was first implemented by Horwitz, Horwitz, and Cope (1986). Furthermore, we need to acknowledge the importance of the role of test anxiety in language classrooms.
Lee, 2016	Situation-specific anxiety means the probability of becoming anxious in a certain situation (MacIntyre & Gardner, 1994), for instance, when using a foreign language, as shown by the foreign language reading anxiety scales (FLRAS) and foreign language listening anxiety scales (FLLAS).
Li, 2015	Anxiety is a complex psychological construct consisting of many variables. It is difficult to collapse them all into a single concise definition. Linguists have come up defections from various aspects (Spielberger, 1983; MacIntyre & Gardner, 1994; Madigan et al., 1996; Sellers, 2000).
Liu, 2016	(I) Defined as the ''apprehension experienced when a situation requires the use of a second language with which the individual is not fully proficient'' (MacIntyre and Gardner 1994, p. 5), FLA has been found to be existent in all aspects of SL/FL learning such as listening, speaking, reading and writing and mainly debilitates the learning of a SL/FL (Ewald 2007; Dewaele and Al-Saraj 2015; Dewaele and Tsui 2013; Horwitz et al. 1986; MacIntyre and Gardner 1991, 1994; Marcos-Llina´s and Garau 2009; Tallon 2009). (II) Listening anxiety has been found to exist in SL/FL listening-related tasks due to such factors as incomprehensibility, task difficulty, task unfa- miliarity, and fear of embarrassing outcomes (Elkhafaifi 2005; Samaneh and Noordin 2013).
MacIntyre & Gardner, 1994	Language anxiety can be defined as the feeling oftension and apprehension specifically associated with second language contexts, including speaking, listening, and learning.
MacIntyre, 1995	(I) Social anxiety is defined by "(1) feelings of tension and discomfort, (2) negative self- evaluations, and (3) a tendency to withdraw in the presence of others" (Schwarzer, 1986, p. 1).<br>(II) The cognitive and affective components of anxiety were identified by Liebert and Morris (1967) as "worry" and "emotionality" respectively. Sarason (1986) defined worry as ". . . distressing preoccupations and concerns about impending events" (p. 21). This preoccupation often takes the form of self-related cognition, which is seldom beneficial for task performance.<br>(III) For some students, this is a frequent course of events, and anxiety becomes reliably associated with any situation involving the second language.
Mills et al., 2006	Bandura (1997) defined anxiety as "a state of anticipatory apprehension over possible deleterious happenings" (p. 137).
Mills et al.,	French anxiety in reading and listening is the state ofanticipatory apprehension

2007	related to listening and reading in French. Bandura (1997) defined anxiety as "a state of anticipatory apprehension over possible deleterious happenings" (p. 137). Individuals experiencing anxiety embody apprehension and avoidant behavior that often interfere with performance in everyday life as well as in academic situations.
Moghadam et al., 2015	Foreign language listening anxiety was seen as a distinct type of situation-specific anxiety.
Mohammadi Golchi, 2012	(I) MacIntyre (1995) believed that listeners in L2 worry about misunderstanding or non-understanding, and they fear embarrassing outcomes.<br>(II) During listening process, different factors may cause uneasiness and tension for language learners and result in poor listening.
Movahed, 2014	Bekleyen (2009) defines foreign language listening anxiety (FLLA) as a kind of anxiety aroused in situations which need listening.
Namaziandost et al., 2018	(I) MacIntyre (1995) states, L2 listeners are anxious about mis/non-understanding and also worry about embarrassing results.<br>(II) As an affective factor which might be individually based, test-taking anxiety has recently been studied in different contexts.
Niimoto, 2021	Foreign language anxiety is defined as "the feeling of tension and apprehension specifically associated with second-language contexts, including speaking, listening and learning" (MacIntyre, & Gardner, 1994, p. 284). Listening anxiety over a foreign language is one such a feeling that learners experience while listening (Yamauchi, 2014b).
Noro, 2006	Noro (2005a) introduced the psychological construct of listening stress as an alternative to listening anxiety in the affective domain of the listening process. Listening stress was defined as "psychological inhibition debilitating listening comprehension which L2/FL learners experience in the face of listening tasks that they perceive to be too difficult" (Noro, 2005b, p. 138).
Noro, 2010	The conceptualization of "listening stress" was based on Lazarus and Folkman's (1984) psychological stress theory, defining it as "psychological inhibition debilitating listening comprehension which L2/FL learners experience in the face of listening tasks that they perceive to be too difficult" (Noro, 2005b, p. 138).
Nurkhamidah, 2020	Listener not only receive the information from speakers, but they must process the meaning of the speakers' utterances. Comprehend people's utterance is not easy. Students in university find many problems in listening comprehension (Hamouda, 2013). This difficulty and complexity cause anxiety among second or foreign language learners (Graham, 2006).
Otair & Abd Aziz, 2017	Language anxiety is defined as "the feeling of tension and apprehension, specifically associated with second language contexts, including speaking, listening, and learning" (Maclntyre and Gardner, 1994, p.24).
Pae, 2013	It has been suggested that anxiety interferes with FL learning (Horwitz et al. 1986; MacIntyre and Gardner 1989; Price 1991; Saito and Samimy 1996), and anxious FL learners tend to show physiological as well as psycholinguistic

	symptoms.
Pan, 2016	Anxiety is defined as "the subjective feeling of tension, apprehension, nervousness, and worry associated with an arousal of the autonomic nervous system" (Spielberger, 1983, p. 1), in the eyes of Arnold and Brown (1999, p. 8), "is associated with negative feelings such as uneasiness, frustration, self-doubt, apprehension and tension."
Polat & Eristi, 2019	Foreign language listening anxiety can be defined as "feelings of apprehension, restlessness, tension, uneasiness and fear, experienced by language learners, stemming from actions required before and during the listening activity as well as other various stimuli" (Polat & Erişti, 2018).
Rahimi & Soleymani, 2015	This type of anxiety which is associated with listening tasks is called listening anxiety.
Ranto Rozak et al., 2019	It was identified that the characteristics of spoken texts such as the main ideas missing, disability to catch the keywords, the speed rate, and little time to process the aural input are the main variables of their FLLA (Chang, 2010).
Rezaabadi, 2016	According to MacIntyre and Gardner (1994), students suffering from FL anxiety are tense and apprehensive, especially when participating in listening and speaking activities in the second language.
Serraj & Noordin, 2013	MacIntyre (1995) explained the reason for such an anxiety is that learners often worry about misunderstanding what they listen to and the fear of being embarrassed by interpreting the message wrongly (Chastain, 1979).
Tsai, 2013	Vogely (1998) showed many potential sources of listening comprehension anxiety based on students' reports: input that is not clear or that is given too fast, and students' belief that they have to make sense of every single word.
Vafaee & Suzuki, 2019	(I) L2 anxiety is defined as the latter, situation-specific anxiety, meaning "the feeling of tension and apprehension specifically associated with second language context, including speaking, listening and learning" (MacIntyre & Gardner, 1994, p. 284).<br>(II) According to Scarcella and Oxford (1992), listening L2 anxiety usually occurs when learners feel they are faced with a difficult and unfamiliar L2 listening task. L2 listening anxiety increases when listeners are under the false impression that to complete a listening task, usually in a testing situation, they must understand every single word they hear (Vogely, 1998).
Valizadeh & Alavinia, 2013	MacIntyre and Gardner (1994) delineate foreign language learning anxiety as "the feeling of tension and apprehension especially associated with second language context, including speaking, listening, reading and writing" (p. 288).
Vogely，1998	According to Scarcella and Oxford (1992), listening anxiety occurs when students feel they are faced with a task that is too difficult or unfamiliar to them. This anxiety is exacerbated if the listeners are under the false impression that they must understand every word they hear.
Wang & Cha, 2019	FLLA is conceptualized as situation-specific and refers to the tendency that FL listeners become anxious in listening-related tasks (Liu, 2016; Zhang, 2013).
Wang, 2010	(I) Anxiety is an affective factor in listening comprehension;

	(II) FLLA plays a very important role because the anticipation of foreign language use in receiving information can provoke anxiety.
Wang, 2016	Anxiety is an important affective factor and should not be overlooked, because the anticipation of foreign language use in receiving information can provoke anxiety, especially anxious students might misunderstand linguistic structures or infer meaning from context for fear of making mistakes and losing face.
Xu & Huang, 2018	L2 listening comprehension can be broken by two components: foreign language listening anxiety (shortened as listening anxiety thereafter) and test anxiety (Vandergrift 2015).
Xu, 2011	The anxiety that arises during the listening process often springs form what Joiner (1986) calls a negative "listening self-concept," that is, a low level of self-confidence in the area of listening.
Xu, 2017	Listening anxiety, referring to the upset or nervousness caused by the listening comprehension.
Yamauchi, 2014a	While listening, most learners worry about the nature of the input (Vogely, 1998).
Yamauchi, 2014b	Foreign language anxiety is defined as "feeling of tension and apprehension specifically associated with second-1anguage contexts, including speaking, listening, and learning" (Macintyre & Gardner, 1994, p. 284). Foreign language listening anxiety, which this study addresses, is such a feeling that learners experience while listening.
Yang, 2010	The inclusion of somatic anxiety was based on the conceptualization of anxiety from Craft, Magyar, Becker and Feltz (2003), and it refers to one's perception of the physiological effects of the anxiety experience, as reflected in increased autonomic arousal and unpleasant feeling states such as nervousness and tension.
Yassin & Razak, 2017	FLCAS are speaking anxiety which includes two factors, namely communication apprehension and fear of negative evaluation, and listening anxiety which includes communication apprehension.
Zhai, 2015	In the 1970s, some researchers began to realize and acknowledge the existence of listening anxiety in foreign language learning. Wheels (1975) described listening anxiety as "receiver's apprehension— the fear of misinterpreting, inadequately processing or not being able to adjust psychologically to messages sent by others."
Zhang, 2013	FL listening anxiety refers to FL anxiety that is associated specifically with FL listening situations.
